# Supplementary material for: LLM-generated messages can persuade humans on policy issues
Source: Nat Commun. 2025 Jul 1;16:6037. doi: 10.1038/s41467-025-61345-5 (PMC12215518; doi:10.1038/s41467-025-61345-5)
Supplement: Supplementary file 1 — Supplementary Information [file 41467_2025_61345_MOESM1_ESM.pdf]

Supplementary Information

for

LLM-generated messages can persuade humans on policy issues

Authors: Hui Bai[1,2], Jan G. Voelkel[1,3,4], Shane Muldowney[1], Johannes C. Eichstaedt[5], Robb Willer[1,3]

Affiliation:

[1]: Polarization and Social Change Lab, Stanford University, Stanford, CA, USA

[2]: Political Belief Lab, Minnetonka, MN, USA

[3]: Department of Sociology, Stanford University, Stanford, CA, USA

[4]: Brooks School of Public Policy, Cornell University, Ithaca, NY, USA.

[5]: Department of Psychology & Institute for Human-Centered AI, Stanford University, Stanford, CA, USA

TABLE OF CONTENTS

Supplementary Table 1a. Study 1 Results: Predicting Support for a Smoking Ban by Condition 4

Supplementary Table 1b. Study 1 Results: Predicting Change in Support for a Smoking Ban by Condition 4

Supplementary Table 2a. Study 2 Results: Predicting Support for an Assault Weapon Ban by Condition 5

Supplementary Table 2b. Study 2 Results: Predicting Change in Support for an Assault Weapon Ban by Condition 5

Supplementary Table 3a. Study 3 Results: Predicting Support for Policy by Condition 6

Supplementary Table 3b. Study 3 Results: Predicting Change in Support for Policy by Condition 6

Supplementary Methods

Measures 7

Descriptive Statistics for Studies 1, 2, and 3 14

Analyses of Other Post-Treatment Measures 16

Supplementary Table 4. Studies 1 and 2 Results: Predicting Support Evaluation of the Policy by Condition 17

Supplementary Table 4 (continue). Studies 1 and 2 Results: Predicting Support Evaluation of the Policy by Condition 18

Author Perception Analysis 20

Supplementary Table 5. Comparing Perceptions of the AI and Human Authors 21

Supplementary Table 5 (continue). Comparing Perceptions of the AI and Human Authors 22

Analyses of the Linguistic Features of LLM and Human Messages 23

Supplementary Table 6. Linguistic Features of LLM and Human Messages 23

Supplementary Table 6 (continue). Linguistic Features of LLM and Human Messages 24

Supplementary Table 6 (continue). Linguistic Features of LLM and Human Messages 25

Moderators of the Persuasion Effects 26

Supplementary Table 7. Studies 1 and 2 Results: Moderating Effects of Party Identity and Pre-Treatment Support 27

Mediators of the Persuasion Effects 28

Supplementary Table 8a. Mediation Analysis (Mediators Tested Separately) 29

Supplementary Table 8b. Mediation Analysis (Mediators Tested Simultaneously) 29

Correlations Between Message Features and Message Persuasiveness 31

Supplementary Table 9. Correlation between Perceived Features of the Messages (at Participant Level) in Each Condition and Change in Support 31

Supplementary Table 10. Correlation between Features of the Messages (at Message Level) in Each Condition and Change in Support 32

Supplementary Table 10 (continue). Correlation between Features of the Messages (at Message Level) in Each Condition and Change in Support 33
Supplementary Table 10 (continue). Correlation between Features of the Messages (at Message Level) in Each Condition and Change in Support 34
Supplementary Table 10 (continue). Correlation between Features of the Messages (at Message Level) in Each Condition and Change in Support 35
Comparison of GPT 3 and GPT 4 Messages 36
Supplementary Table 11. Comparison of the Linguistic Features of GPT 3 and GPT4 37
Supplementary Table 11 (continue). Comparison of the Linguistic Features of GPT 3 and GPT4 38
Supplementary Table 11 (continue). Comparison of the Linguistic Features of GPT 3 and GPT4 39
Supplementary Table 11 (continue). Comparison of the Linguistic Features of GPT 3 and GPT4 40
Author Identity Tabulation 41
Participants' Change in Policy Support by Condition Across Studies 42
Supplementary Figure 1 42
Different Perceptions of Human and AI Authors in Studies 1 and 2 43
Supplementary Figure 2 43
Distribution of the Messages' Persuasiveness in Studies 1 and 2 44
Supplementary Figure 3 44
Distribution of Pre-Treatment Levels of Support for Policies 45
Supplementary Figure 4 46
Supplementary Figure 5 46
Supplementary Figure 6 47
Study 3's Issue-Specific Results 48
Covariate Balance Checks 49

Supplementary Discussion

Additional Literature 50
Supplementary Table 12: Summary of Related Research 50
Assumption Checks 57
Supplementary Table 13. Model Re-Estimated Using Huber-White Estimators 58
References 59

Supplementary Table 1a. Study 1 Results: Predicting Support for a Smoking Ban by Condition

		b	S.E.	*p*	95% CI	η² (in %)	β
Model 1	(Intercept)	0.56	0.89	0.528	[-1.19 2.31]		
	Pre-treatment DV	1.00	0.01	<.001	[0.97 1.02]	87.37	0.93
	Human-in-the-Loop condition	5.04	0.91	<.001	[3.26 6.82]	2.52	0.07
	AI condition	3.62	0.87	<.001	[1.92 5.32]	1.43	0.05
	Human condition	3.36	0.86	<.001	[1.67 5.05]	1.25	0.05
	F(4,1198) = 2090.97, *R*-squared = 0.87, adjusted *R*-squared = 0.87						
Model 2	(Intercept)	3.89	1.05	<.001	[1.83 5.94]		
	Pre-treatment DV	1.00	0.01	<.001	[0.97 1.02]	84.98	0.92
	Human-in-the-Loop condition	1.68	0.99	0.089	[-0.26 3.62]	0.32	0.02
	AI condition	0.26	0.95	0.787	[-1.60 2.12]	0.01	0.00
	F(3,892) = 1692.5, *R*-squared = 0.85, adjusted *R*-squared = 0.85						
Model 3	(Intercept)	3.71	1.24	0.003	[1.27 6.15]		
	Pre-treatment DV	1.00	0.02	<.001	[0.97 1.04]	85.91	0.93
	Human-in-the-Loop condition	1.45	0.96	0.131	[-0.43 3.34]	0.40	0.02
	F(2,573) = 1750.66, *R*-squared = 0.86, adjusted *R*-squared = 0.86						

Note. All models are linear regression models without adjustments. Model 1's reference group is the Control condition. Model 2's reference group is the Human condition (the Control condition participants are excluded). Model 3's reference group is the AI Condition (the Control and Human condition participants are both excluded). S.E. refers to standard error of the estimates. All results are two-tailed.

Supplementary Table 1b. Study 1 Results: Predicting Change in Support for a Smoking Ban by Condition

		b	S.E.	*p*	95% CI	η² (in %)	β
Model 1	(Intercept)	0.30	0.62	0.623	[-0.90 1.51]		
	Human-in-the-Loop condition	5.04	0.91	<.001	[3.27 6.82]	2.52	0.19
	AI condition	3.60	0.87	<.001	[1.90 5.30]	1.42	0.14
	Human condition	3.37	0.86	<.001	[1.68 5.06]	1.26	0.14
	F(3,1199) = 11.51, *R*-squared = 0.03, adjusted *R*-squared = 0.03						
Model 2	(Intercept)	3.67	0.66	<.001	[2.37 4.97]		
	Human-in-the-Loop condition	1.68	0.99	0.090	[-0.26 3.62]	0.32	0.06
	AI condition	0.24	0.94	0.801	[-1.62 2.09]	0.01	0.01
	F(2,893) = 1.63, *R*-squared = 0, adjusted *R*-squared = 0						
Model 3	(Intercept)	3.91	0.65	<.001	[2.64 5.18]		
	Human-in-the-Loop condition	1.44	0.96	0.133	[-0.44 3.32]	0.39	0.06
	F(1,574) = 2.27, *R*-squared = 0, adjusted *R*-squared = 0						

Note. All models are linear regression models without adjustments. Model 1's reference group is the Control condition. Model 2's reference group is the Human condition (the Control condition participants are excluded). Model 3's reference group is the AI Condition (the Control and Human condition participants are both excluded). S.E. refers to standard error of the estimates. All results are two-tailed.

Supplementary Table 2a. Study 2 Results: Predicting Support for an Assault Weapon Ban by Condition

		b	S.E.	*p*	95% CI	η^2 (in %)	β
Model 1	(Intercept)	-2.41	0.51	<.001	[-3.40 -1.41]		
	Pre-treatment DV	1.05	0.01	<.001	[1.04 1.06]	93.32	0.96
	Human-in-the-Loop condition	2.35	0.57	<.001	[1.23 3.46]	0.84	0.03
	AI condition	1.81	0.57	0.002	[0.69 2.93]	0.49	0.02
	Human condition	2.35	0.57	<.001	[1.23 3.47]	0.84	0.03
	F(4,2011) = 7085.67, *R*-squared = 0.93, adjusted *R*-squared = 0.93						
Model 2	(Intercept)	-0.41	0.62	0.511	[-1.63 0.81]		
	Pre-treatment DV	1.05	0.01	<.001	[1.04 1.07]	92.21	0.96
	Human-in-the-Loop condition	0.01	0.62	0.984	[-1.20 1.23]	0.00	0.00
	AI condition	-0.52	0.62	0.403	[-1.74 0.70]	0.05	-0.01
	F(3,1501) = 5944.03, *R*-squared = 0.92, adjusted *R*-squared = 0.92						
Model 3	(Intercept)	-1.45	0.67	0.031	[-2.76 -0.14]		
	Pre-treatment DV	1.06	0.01	<.001	[1.05 1.08]	92.47	0.96
	Human-in-the-Loop condition	0.52	0.62	0.403	[-0.70 1.74]	0.07	0.01
	F(2,996) = 6117.26, *R*-squared = 0.92, adjusted *R*-squared = 0.92						

Note. All models are linear regression models without adjustments. Model 1's reference group is the Control condition. Model 2's reference group is the Human condition (the Control condition participants are excluded). Model 3's reference group is the AI Condition (the Control and Human condition participants are both excluded). S.E. refers to standard error of the estimates. All results are two-tailed.

Supplementary Table 2b. Study 2 Results: Predicting Change in Support for an Assault Weapon Ban by Condition

		b	S.E.	*p*	95% CI	η^2 (in %)	β
Model 1	(Intercept)	-0.01	0.41	0.990	[-0.80 0.79]		
	Human-in-the-Loop condition	2.53	0.58	<.001	[1.39 3.66]	0.94	0.12
	AI condition	1.92	0.58	0.001	[0.79 3.06]	0.55	0.09
	Human condition	2.65	0.58	<.001	[1.52 3.78]	1.04	0.12
	F(3,2012) = 9.05, *R*-squared = 0.01, adjusted *R*-squared = 0.01						
Model 2	(Intercept)	2.65	0.44	<.001	[1.78 3.52]		
	Human-in-the-Loop condition	-0.13	0.63	0.841	[-1.36 1.11]	0.00	-0.01
	AI condition	-0.73	0.63	0.249	[-1.96 0.51]	0.09	-0.03
	F(2,1507) = 0.74, *R*-squared = 0, adjusted *R*-squared = 0						
Model 3	(Intercept)	1.92	0.45	<.001	[1.03 2.80]		
	Human-in-the-Loop condition	0.60	0.64	0.344	[-0.65 1.85]	0.09	0.03
	F(1,1000) = 0.89, *R*-squared = 0, adjusted *R*-squared = 0						

Note. All models are linear regression models without adjustments. Model 1's reference group is the Control condition. Model 2's reference group is the Human condition (the Control condition participants are excluded). Model 3's reference group is the AI Condition (the Control and Human condition participants are both excluded). S.E. refers to standard error of the estimates. All results are two-tailed.

Supplementary Table 3a. Study 3 Results: Predicting Support for Policy by Condition

		b	S.E	*p*	95% CI	η^2 (in %)	β
Overall model	(Intercept)	1.17	0.66	0.075	[-0.12 2.47]		
	Pre-treatment DV	0.99	0.01	<.001	[0.98 1.01]	92.35	0.96
	AI condition	3.15	0.43	<.001	[2.31 3.99]	3.26	0.05
	Paid parental leave	-0.44	0.61	0.478	[-1.64 0.77]	0.03	-0.01
	Child tax credit	0.69	0.61	0.256	[-0.50 1.88]	0.08	0.01
	Automatic voter registration	-1.88	0.60	0.002	[-3.06 -0.69]	0.60	-0.03
	F(5,1604)=3950.63, R-squared=0.92, adjusted R-squared=0.92						
Carbon tax model	(Intercept)	0.30	0.90	0.740	[-1.47 2.06]		
	Pre-treatment DV	1.00	0.01	<.001	[0.98 1.03]	94.82	0.97
	AI condition	3.74	0.71	<.001	[2.34 5.14]	6.48	0.06
	F(2,398) = 3654.6, *R*-squared = 0.95, adjusted *R*-squared = 0.95						
Child tax credit model	(Intercept)	1.49	1.28	0.243	[-1.01 4.00]		
	Pre-treatment DV	0.98	0.02	<.001	[0.95 1.02]	88.91	0.94
	AI condition	5.03	0.98	<.001	[3.11 6.96]	6.21	0.09
	F(2,399)=1605.39, R-squared=0.89, adjusted R-squared=0.89						
Paid parental leave model	(Intercept)	1.10	1.14	0.334	[-1.13 3.34]		
	Pre-treatment DV	0.99	0.01	<.001	[0.97 1.02]	92.94	0.96
	AI condition	2.39	0.80	0.003	[0.82 3.96]	2.26	0.04
	F(2,387) = 2562.43, *R*-squared = 0.93, adjusted *R*-squared = 0.93						
Automatic voter registration model	(Intercept)	0.19	1.17	0.869	[-2.10 2.49]		
	Pre-treatment DV	0.99	0.01	<.001	[0.97 1.02]	92.58	0.96
	AI condition	1.45	0.90	0.107	[-0.31 3.21]	0.63	0.02
	F(2,414)=2582.17, R-squared=0.93, adjusted R-squared=0.93						

Note. All models are linear regression models without adjustments. S.E. refers to standard error of the estimates. All results are two-tailed.

Supplementary Table 3b. Study 3 Results: Predicting Change in Support for Policy by Condition

		b	S.E	*p*	95% CI	η^2 (in %)	β
Overall model	(Intercept)	0.81	0.48	0.093	[-0.13 1.75]		
	AI condition	3.15	0.43	<.001	[2.31 3.99]	3.26	0.18
	Paid parental leave	-0.49	0.61	0.427	[-1.68 0.71]	0.04	-0.02
	Child tax credit	0.70	0.61	0.246	[-0.49 1.89]	0.08	0.03
	Automatic voter registration	-1.92	0.60	0.001	[-3.09 -0.74]	0.63	-0.1
	F(4,1605)=18.62, *R*-squared=0.04, adjusted *R*-squared=0.04						
Carbon tax model	(Intercept)	0.51	0.50	0.313	[-0.48 1.50]		
	AI condition	3.74	0.71	<.001	[2.34 5.14]	6.48	0.25
	F(1,399) = 27.64, *R*-squared = 0.06, adjusted *R*-squared = 0.06						
Child tax credit model	(Intercept)	0.55	0.69	0.429	[-0.82 1.91]		
	AI condition	5.06	0.98	<.001	[3.13 6.98]	6.25	0.25
	F(1,400) = 25.55, *R*-squared = 0.06, adjusted *R*-squared = 0.06						
Paid parental leave model	(Intercept)	0.71	0.56	0.211	[-0.40 1.82]		
	AI condition	2.38	0.80	0.003	[0.81 3.94]	2.25	0.15
	F(1,388) = 8.92, *R*-squared = 0.02, adjusted *R*-squared = 0.02						
Automatic voter registration model	(Intercept)	-0.26	0.64	0.688	[-1.51 1.00]		
	AI condition	1.46	0.90	0.104	[-0.30 3.22]	0.63	0.08
	F(1,415) = 3.42, *R*-squared = 0.01, adjusted *R*-squared = 0.01						

Note. All models are linear regression models without adjustments. S.E. refers to standard error of the estimates. All results are two-tailed.

Supplementary Methods

Measures

Study 1

Attention Check

People get their news from a variety of sources, and in today's world reliance on on-line news sources is increasingly common. We want to know how much of your news consumption comes from on-line sources. We also want to know if people are paying attention to the question. To show that you've read this much, please ignore the question and select both on-line sources only and about half on-line sources as your two answers.

About how much of your news consumption comes from on-line sources? Please include print newspapers that you read on-line (e.g., washingtonpost.com) as on-line sources.

- On-line sources only.
- Mostly on-line sources, with some television and print news.
- About half on-line sources.
- Mostly television or print news, with some on-line sources.
- Television or print news only.

Dependent Variable Measure

Please indicate your level of agreement with the following statements:

- We should implement a total smoking ban in all public places.
- A total smoking ban in all public places is a bad idea (reverse-coded).
- A total smoking ban in all public places would have good consequences.
  [0 = Strongly disagree; 100 = Strongly agree]

- Do you support or oppose a total smoking ban in all public places?
  [0 = Strongly oppose; 100 = Strongly support]

- If there was a referendum tomorrow about a total smoking ban in all public places, how likely is it that you would vote in favor of a smoking ban?
  [0 = 0% chance, definitely would not; 100 = 100% chance, definitely would]

Other Post-Treatment Measures

How much do you agree with the following statements? (0 = Strongly disagree; 100 = Strongly agree):

- A total smoking ban is a smart idea.
- A total smoking ban is a logical idea.
- A total smoking ban is an empathetic idea.
- A total smoking ban is a compassionate idea.
- A total smoking ban is a moral idea.
- A total smoking ban is an ethical idea.

How well do the following describe the author of the message? (0 = Not at all; 100 = A great deal):

- Smart
- Intelligent
- Compassionate
- Empathetic
- Warm
- Cold
- Pushy
- Angry
- Logical
- Moral
- Ethical
- Factual
- Well-informed.

Study 2

Attention Check

Participants in Study 2 responded to the same attention check as in Study 1.

Dependent Variable Measure

Please indicate your level of agreement with the following statements:

- We should implement an assault weapon ban..
- An assault weapon ban is a bad idea (reverse-coded).
- An assault weapon ban would have good consequences.
  [0 = Strongly disagree; 100 = Strongly agree]

- Do you support or oppose an assault weapon ban?
  [0 = Strongly oppose; 100 = Strongly support]

- If there was a referendum tomorrow about an assault weapon ban, how likely is it that you would vote in favor of an assault weapon ban?
  [0 = 0% chance, definitely would not; 100 = 100% chance, definitely would]

Other Post-Treatment Measures

How much do you agree with the following statements? (0 = Strongly disagree; 100 = Strongly agree):

- An assault weapon ban is a smart idea.
- An assault weapon ban is a logical idea.
- An assault weapon ban is an empathetic idea.
- An assault weapon ban is a compassionate idea.
- An assault weapon ban is a moral idea.
- An assault weapon ban is an ethical idea.

How well do the following describe the author of the message? (0 = Not at all; 100 = A great deal):

- Used a lot of facts and evidence
- Was very well-informed
- Referenced their personal experiences
- Told a story
- Described vivid scenarios
- Smart

- Intelligent
- Empathetic
- Warm
- Assertive
- Angry
- Logical
- Moral
- Ethical
- Creative
- Original
- Authentic
- Genuine
- Has a unique voice
- Interesting

Based on the message, how would you describe the author of the message? Did you have any questions about the author? [open-ended question]:

The message is most likely written by which of the following?:

- An adult person.
- A group of people
- An expert on the topic
- An artificial intelligence program
- An intelligent adolescent
- An elementary school-age child
- Other (please specific)___.

Study 3

Attention check

Participants in Study 3 responded to the same attention check as in Studies 1 and 2.

Dependent Variable Measure

[Carbon tax]

Please answer the following questions about a carbon tax. A carbon tax is a tax imposed on businesses organizations that produce or consume fossil fuels (such as coal, oil, and natural gas) that are intended to reduce emissions of carbon dioxide and other greenhouse gasses.

Please indicate your level of agreement with the following statements:

- The U.S. federal government should impose a carbon tax
- A federal carbon tax is a bad idea
- A federal carbon tax would have good consequences
  [0 = Strongly disagree; 0 = Neither agree nor disagree; 100 = Strongly agree]

- Do you support or oppose implementing a federal carbon tax?
  [0 = Strongly oppose; 50 = Neither support nor oppose; 100 = Strongly support]

- If there was a referendum tomorrow about a federal carbon tax, how likely would it be that you would vote in favor of it? [0 = Definitely would not vote in favor of it; 100 = Definitely would vote in favor of it]

[Paid parental leave]

Please answer the following questions about the U.S. federal government funding a parental leave program. The federal government funding parental leave refers to the federal government providing financial support to parents who take time off work to care for and bond with newborn children.

Please indicate your level of agreement with the following statements:

- The U.S federal government should fund paid parental leave
- The U.S federal government funding paid parental leave is a bad idea
- The U.S federal government funding paid parental leave would have good consequences
  [0 = Strongly disagree; 0 = Neither agree nor disagree; 100 = Strongly agree]

- Do you support or oppose the U.S. federal government funding paid-parental leave?
  [0 = Strongly oppose; 50 = Neither support nor oppose; 100 = Strongly support]

- If there was a referendum tomorrow about the U.S. federal government funding paid-parental leave, how likely would it be that you would vote in favor of it?
  [0 = Definitely would not vote in favor; 100 = Definitely would vote in favor]

[Child tax credit]
Please answer the following questions about a child tax credit. The Child Tax Credit is a tax credit available to qualifying U.S. taxpayers who are responsible for the care of one or more dependent children under the age of 17.

Please indicate your level of agreement with the following statements:
- The U.S. federal government should increase the child tax credit
- The U.S. federal government increasing the child tax credit is a bad idea
- The U.S. federal government increasing the child tax credit would have good consequences.
  [0 = Strongly disagree; 0 = Neither agree nor disagree; 100 = Strongly agree]

- Do you support or oppose the U.S. federal government increasing the child tax credit?
  [0 = Strongly oppose; 50 = Neither support nor oppose; 100 = Strongly support]

- If there was a referendum tomorrow about the U.S. federal government increasing the child tax credit, how likely would it be that you would vote in favor of it?
  [0 = Definitely would not vote in favor; 100 = Definitely would vote in favor]

[Automatic voter registration]
Please answer the following questions about automatically registering eligible Americans to vote. Automatic voter registration is a system in which eligible citizens are automatically registered to vote when they interact with a government agency, such as the Department of Motor Vehicles.

Please indicate your level of agreement with the following statements:
- Eligible Americans should be automatically registered to vote.

- Automatically registering eligible Americans to vote is a bad idea.
- Automatically registering eligible Americans to vote would have good consequences.
  [0 =Strongly disagree; 0 = Neither agree nor disagree; 100 = Strongly agree]

- Do you support or oppose automatically registering eligible Americans to vote?
  [0 = Strongly oppose; 50 = Neither support nor oppose; 100 = Strongly support]

- If there was a referendum tomorrow about automatically registering eligible Americans to vote, how likely would it be that you would vote in favor of it?
  [0 = Definitely would not vote in favor; 100 = Definitely would vote in favor]

Descriptive Statistics for Studies 1, 2, and 3

Study 1

Descriptively, participants in the LLM condition increased their support for the policy on average by 3.91 points (*SD* = 10.45; pre-treatment: *M* = 62.47, *SD* = 27.38; post-treatment: *M* = 66.38, *SD* = 30.16). Participants in the Human condition increased their support for the policy on average by 3.67 points (*SD* = 12.63; pre-treatment: *M* = 57.59, *SD* = 28.74; post-treatment: *M* = 61.26, *SD* = 30.98). Participants in the Human-in-the-Loop condition increased their support for the policy on average by 5.35 (*SD* = 12.51; pre-treatment: *M* = 58.56, *SD* = 29.05; post-treatment: *M* = 63.90, *SD* = 30.84). Participants in the Control condition' policy support increased on average by 0.30 (*SD* = 6.63; pre-treatment: *M* = 58.88, *SD* = 28.84; post-treatment: *M* = 59.19, *SD* = 29.42).

Study 2

Descriptively, participants in the AI condition increased their average support for the policy by 1.92 points (*SD* = 10.23; pre-treatment: *M* = 52.36, *SD* = 31.60; post-treatment: *M* = 54.28, *SD* = 35.60). Participants in the Human condition increased their average support for the policy by 2.65 points (*SD*= 9.87; pre-treatment: *M* = 56.16, *SD* = 31.57; post-treatment: *M* = 58.80, *SD* = 34.08). Participants in the Curator condition increased their average support for the policy by 2.52 points (*SD* = 9.85; pre-treatment: *M* = 53.61, *SD* = 33.03; post-treatment: *M* = 56.13, *SD* = 35.97). Participants in the Control condition changed their average support for the policy by -0.01 points (*SD* = 6.32; pre-treatment: *M* = 49.91, *SD* = 32.97; post-treatment: *M* = 49.91, *SD* = 34.54).

Study 3

Descriptively, participants in the treatment condition increased their support for the policy by 3.51 (*SD* = 11.05; pre-treatment: *M* = 66.34, *SD* = 30.04; post-treatment *M* = 69.85, *SD* = 31.37). Participants in the control condition increased their support for the policy by 0.37 (*SD* = 5.11; pre-treatment level *M* = 66.54, *SD* = 30.60; post-treatment *M* = 66.91, *SD* = 31.12).

Reviewing the results by topic, among participants who received treatment, participants in the Carbon Tax condition increased their support for the policy by 4.25 (*SD* = 9.25; pre-treatment *M* = 63.21, SD = 30.46; post-treatment *M* = 67.46, *SD* = 31.84). Participants in the Child Tax Credit condition increased their support for the policy by 5.61 (SD = 13.20; pre-treatment *M* = 59.95, *SD* = 28.44; post-treatment *M* = 65.55, *SD* = 29.95). Participants in the Parental Leave Program condition increased their support for the policy by 3.09 (SD = 9.11; pre-treatment *M* = 72.67, *SD* = 27.25; post-treatment *M* = 75.75, *SD* = 28.77). Participants in the Automatic Voter Registration condition increased their support for the policy by 1.20 (*SD* = 11.63; pre-treatment *M* = 69.57, *SD* = 32.12; post-treatment *M* = 70.77, *SD* = 33.78).

Among participants who were in the control condition, participants in the Carbon Tax condition increased their support for the policy by 0.51 (*SD* = 3.96; pre-treatment *M* = 63.08, *SD*

= 30.25; post-treatment *M* = 63.59, *SD* = 30.70). Participants in the Child Tax Credit condition increased their support for the policy by 0.55 (*SD* = 4.22; pre-treatment *M* = 61.41, *SD* = 27.94; post-treatment *M* = 61.96, *SD* = 28.92). Participants in the Parental Leave Program condition increased their support for the policy by 0.71 (SD = 6.36; pre-treatment *M* = 70.83, *SD* = 30.09; post-treatment *M* = 71.53, *SD* = 30.41). Participants in the Automatic Voter Registration condition decreased their support for the policy by 0.26 (*SD* = 5.53; pre-treatment *M* = 70.83, *SD* = 32.84; post-treatment *M* = 70.58, *SD* = 33.29).

Analyses of Other Post-Treatment Measures

As preregistered, we conducted exploratory analyses to examine whether the participants in the LLM, Human, and Human-in-the-Loop conditions rated the policies more favorably than the Control condition. In short, these results are very similar to the results for the main policy support variable reported in the main text. The detailed results can be found in Supplementary Table 4 below (also see osf.io/8yxvr for all supplementary tables).

Supplementary Table 4. Studies 1 and 2 Results: Predicting Support Evaluation of the Policy by Condition

		b		S.E.	*p*	95% CI		η^2 (in %)	β
Smart Model 1	(Intercept)	0.92	ns	0.79	0.244	[-0.63	2.46]		
	Human-in-the-Loop condition	3.89	***	0.75	<.001	[2.42	5.36]	0.83	0.05
	AI condition	2.91	***	0.74	<.001	[1.47	4.36]	0.48	0.04
	Human condition	2.80	***	0.73	<.001	[1.36	4.24]	0.45	0.04
	Pre-treatment DV	1.00	***	0.01	<.001	[0.99	1.02]	81.36	0.90
	Study	-1.84	***	0.54	0.001	[-2.91	-0.78]	0.36	-0.03
	F(5,3210) = 2867.45, *R*-squared = 0.82, adjusted *R*-squared = 0.82								
Smart Model 2	(Intercept)	3.84	***	0.92	<.001	[2.04	5.64]		
	Human-in-the-Loop condition	1.12	ns	0.78	0.154	[-0.42	2.65]	0.08	0.01
	AI condition	0.12	ns	0.77	0.881	[-1.40	1.63]	0.00	0.00
	Pre-treatment DV	1.01	***	0.01	<.001	[0.99	1.03]	79.77	0.89
	Study	-2.35	***	0.66	<.001	[-3.64	-1.05]	0.52	-0.03
	F(4,2395) = 2396.15, *R*-squared = 0.8, adjusted *R*-squared = 0.8								
Smart Model 3	(Intercept)	3.09	**	1.05	0.003	[1.03	5.14]		
	Human-in-the-Loop condition	1.01	ns	0.77	0.188	[-0.50	2.52]	0.11	0.01
	Pre-treatment DV	1.02	***	0.01	<.001	[1.00	1.04]	81.11	0.90
	Study	-2.27	**	0.81	0.005	[-3.84	-0.69]	0.51	-0.03
	F(3,1570) = 2299.71, *R*-squared = 0.81, adjusted *R*-squared = 0.81								
Compassionate Model 1	(Intercept)	8.75	***	1.17	<.001	[6.46	11.05]		
	Human-in-the-Loop condition	3.49	**	1.11	0.002	[1.32	5.67]	0.31	0.05
	AI condition	4.70	***	1.10	<.001	[2.55	6.84]	0.57	0.06
	Human condition	4.59	***	1.09	<.001	[2.45	6.72]	0.55	0.06
	Pre-treatment DV	0.76	***	0.01	<.001	[0.73	0.78]	52.94	0.73
	Study	5.67	***	0.81	<.001	[4.09	7.26]	1.51	0.09
	F(5,3209) = 733.47, *R*-squared = 0.53, adjusted *R*-squared = 0.53								
Compassionate Model 2	(Intercept)	13.74	***	1.31	<.001	[11.17	16.31]		
	Human-in-the-Loop condition	-1.06	ns	1.12	0.343	[-3.25	1.13]	0.04	-0.02
	AI condition	0.11	ns	1.10	0.921	[-2.05	2.27]	0.00	0.00
	Pre-treatment DV	0.76	***	0.01	<.001	[0.73	0.79]	52.20	0.72
	Study	4.96	***	0.94	<.001	[3.11	6.81]	1.14	0.07
	F(4,2395) = 654.84, *R*-squared = 0.52, adjusted *R*-squared = 0.52								
Compassionate Model 3	(Intercept)	12.08	***	1.49	<.001	[9.16	15.00]		
	Human-in-the-Loop condition	-1.13	ns	1.09	0.299	[-3.27	1.01]	0.07	-0.02
	Pre-treatment DV	0.79	***	0.02	<.001	[0.75	0.82]	56.02	0.75
	Study	4.79	***	1.14	<.001	[2.56	7.03]	1.12	0.07
	F(3,1570) = 667.73, *R*-squared = 0.56, adjusted *R*-squared = 0.56								

Note. All models are linear regression models without adjustments. The Study variable is coded as 0 for Study 1, and 1 for Study 2. Model 1's reference group is the Control condition. Model 2's reference group is the Human condition (the Control condition participants are excluded). Model 3's reference group is the AI Condition (the Control and Human condition participants are both excluded). S.E. refers to standard error of the estimates. All results are two-tailed.

Supplementary Table 4 (continue). Studies 1 and 2 Results: Predicting Support Evaluation of the Policy by Condition

		b		S.E.	*p*	95% CI		η² (in %)	β
Ethical Model 1	(Intercept)	4.94	***	1.07	<.001 [	2.84	7.05]		
	Human-in-the-Loop condition	2.87	**	1.02	0.005 [	0.87	4.87]	0.25	0.04
	AI condition	3.11	**	1.01	0.002 [	1.14	5.08]	0.30	0.04
	Human condition	3.15	**	1.00	0.002 [	1.19	5.11]	0.31	0.04
	Pre-treatment DV	0.87	***	0.01	<.001 [	0.85	0.90]	64.05	0.80
	Study	2.59	***	0.74	0.001 [	1.13	4.04]	0.38	0.04
	F(5,3210) = 1156.17, *R*-squared = 0.64, adjusted *R*-squared = 0.64								
Ethical Model 2	(Intercept)	7.61	***	1.21	<.001 [	5.24	9.97]		
	Human-in-the-Loop condition	-0.27	ns	1.03	0.791 [	-2.29	1.75]	0.00	0.00
	AI condition	-0.04	ns	1.01	0.970 [	-2.03	1.95]	0.00	0.00
	Pre-treatment DV	0.88	***	0.01	<.001 [	0.85	0.91]	63.66	0.80
	Study	2.62	**	0.87	0.003 [	0.91	4.32]	0.38	0.04
	F(4,2396) = 1051.52, *R*-squared = 0.64, adjusted *R*-squared = 0.64								
Ethical Model 3	(Intercept)	6.51	***	1.37	<.001 [	3.82	9.21]		
	Human-in-the-Loop condition	-0.18	ns	1.01	0.859 [	-2.15	1.79]	0.00	0.00
	Pre-treatment DV	0.91	***	0.02	<.001 [	0.88	0.94]	66.47	0.82
	Study	1.90	ns	1.05	0.070 [	-0.16	3.97]	0.21	0.03
	F(3,1571) = 1046.05, *R*-squared = 0.67, adjusted *R*-squared = 0.67								

Note. All models are linear regression models without adjustments. The Study variable is coded as 0 for Study 1, and 1 for Study 2. Model 1's reference group is the Control condition. Model 2's reference group is the Human condition (the Control condition participants are excluded). Model 3's reference group is the AI Condition (the Control and Human condition participants are both excluded). S.E. refers to standard error of the estimates. All results are two-tailed.

Procedure

We measured several composites capturing different aspects of favorability toward the policy. The *evaluation of the policy as smart* was measured with an average of two items: "A total smoking ban is a smart idea" and "A total smoking ban is a logical idea" in Study 1, and "An assault weapon ban is a smart idea" and "An assault weapon ban is an logical idea" in Study 2 (α = .96). The *evaluation of the policy as empathic* was measured with an average of two items: "A total smoking ban is an empathetic idea" and "A total smoking ban is a compassionate idea" in Study 1, and "An assault weapon ban is a empathetic idea" and "An assault weapon ban is a compassionate idea" in Study 2 (α = .97). The *evaluation of the policy as moral* was measured with an average of two items: "A total smoking ban is a moral idea" and "A total smoking ban is an ethical idea" in Study 1, and "An assault weapon ban is a moral idea" and "An assault weapon ban is an ethical idea" in Study 2 (α = .97). All items were preceded by the instruction"How much do you agree with the following statements?" (0 = Strongly disagree; 100 = Strongly agree).

Analysis

We combined the data from Studies 1 and 2. Like the analyses for the main policy support variables, we ran three regression models for each additional outcome. In the first

regression, the outcome was regressed on the dummy-coded variables for “Human-in-the-Loop”, “LLM”, and “Human” conditions (all contrasted with the neutral Control condition, which was the reference category) while controlling for the pre-treatment measure of the policy support variable and a dummy variable for Study.

In the second regression, the outcome was regressed on the dummy-coded variables for “Human-in-the-Loop” and “LLM” conditions (both contrasted with the “Human” condition). The model excluded the Control group participants while controlling for the pre-treatment measure of the smoking ban support variable.

In the third regression, the outcome was regressed on the dummy-coded variable for the “Human-in-the-Loop” condition (contrasted with the “LLM” condition, which was the reference category). The model excluded the Human group and the Control group participants while controlling for the pre-treatment measure of the smoking ban support variable.

Results

Smart. LLM- and human-generated messages caused participants to evaluate the policy as smarter. Participants in the LLM ($b = 2.91$, CI = [1.47, 4.36], $p < .001$), Human ($b = 2.80$, CI = [1.36, 4.24], $p < .001$), and Human-in-the-Loop ($b = 3.89$, CI = [2.42, 5.36], $p < .001$) conditions all rated the policy as smarter than participants in the Control condition. Participants in the LLM ($b = 0.12$, CI = [-1.40, 1.63], $p = 0.881$, BF01 = 48.44) and Human-in-the-Loop condition ($b = 1.12$, CI = [-0.42, 2.65], $p = 0.154$, BF01 = 15.56) rated the policy similarly smart compared to participants in the Human condition. Furthermore, participants in the Human-in-the-Loop condition rated the policy to be similarly smart compared to participants in the LLM condition ($b = 1.01$, CI = [-0.50, 2.52], $p = 0.188$, BF01 = 16.66).

Compassionate. LLM- and human-generated messages caused participants to evaluate the policy as more compassionate. Participants in the LLM ($b = 4.70$, CI = [2.55, 6.84], $p < .001$), Human ($b = 4.59$, CI = [2.45, 6.72], $p < .001$), and Human-in-the-Loop ($b = 3.49$, CI = [1.32, 5.67], $p = 0.002$) conditions all rated the policy as more compassionate than participants in the Control condition. Participants in the LLM ($b = 0.11$, CI = [-2.05, 2.27], $p = 0.921$, BF01 $\gg$10,000) and Human-in-the-Loop condition ($b = -1.06$, CI = [-3.25, 1.13], $p = 0.343$, BF01 = 25.29) rated the policy similarly compassionate compared to participants in the Human condition. Furthermore, participants in the Human-in-the-Loop condition rated the policy to be similarly compassionate compared to participants in the LLM condition ($b = -1.13$, CI = [-3.27, 1.01], $p = 0.299$, BF01 = 23.10).

Ethical. LLM- and human-generated messages caused participants to evaluate the policy as more ethical. Participants in the LLM ($b = 3.11$, CI = [1.14, 5.08], $p = 0.002$), Human ($b = 3.15$, CI = [1.19, 5.11], $p = 0.002$), and Human-in-the-Loop ($b = 2.87$, CI = [0.87, 4.87], $p = 0.005$) conditions all rated the policy as more ethical than participants in the Control condition. Participants in the LLM ($b = -0.04$, CI = [-2.03, 1.95], $p = 0.970$, BF01 $\gg$10,000) and Human-in-the-Loop condition ($b = -0.27$, CI = [-2.29, 1.75], $p = 0.791$, BF01 = 38.20) rated the policy similarly ethical compared to participants in the Human condition. Furthermore, participants in the Human-in-the-Loop condition rated the policy to be similarly ethical compared to participants in the LLM condition ($b = -0.18$, CI = [-2.15, 1.79], $p = 0.859$, BF01 = 39.07).

Author Perception Analyses

We examined differences in the perceptions of the author of messages when the messages were generated by LLM versus human authors.

Procedure

Participants were asked to rate the author using the question "How well do the following describe the author of the message? (0 = not at all; 100 = a great deal)" on a number of traits that we pre-registered as exploratory measures. Traits asked only in Study 1 are: pushy, compassionate, cold, and factual. Traits asked only in Study 2 are: used a lot of facts and evidence, referenced their personal experiences, told a story, described vivid scenarios, assertive, creative, original, authentic, genuine, has a unique voice, and interesting. Traits asked in both studies are: was very well-informed, smart, intelligent, empathetic, warm, angry, logical, moral, and ethical.

We used several composites and several individual items as outcomes for these analyses. The composites we created from several highly correlated items are: a *smart* composite (scaled from "smart", and "intelligent"; $\alpha = .99$), a *factual* composite (scaled from "was very well-informed", "factual", and "used a lot of facts and evidence"; $\alpha = .95$), a *moral* composite (scaled from "moral" and "ethical"; $\alpha = .97$), a *warm* composite (scaled from "empathetic", "compassionate", reverse-coded "cold" and "warm" in Study 1 and "empathetic" and "warm" in Study 2; $\alpha = .86$), an *authentic* composite (scaled from "authentic" and "genuine"; $\alpha = .96$), a *unique* composite (scaled from "original" and "has a unique voice"; $\alpha = .86$), a *story-telling* composite (scaled from "referenced their personal experiences", "told a story", and "described vivid scenarios"; $\alpha = .79$), and an *assertive* (measured with "pushy" in Study 1 and "assertive" in Study 2). Note that the factual composite does not include "Used a lot of facts and evidence" in Study 1 and "factual" in Study 2. The individual items we used without scaling are: angry, creative, interesting, cold, compassionate, and logical.

Analysis Strategy

To investigate differences in the perceptions of the LLM author and the human authors, we regressed the outcomes on participants' pre-treatment level of support, experimental condition(1 = AI, 0 = human), and study (only for models that predict variables that appeared in both studies).

Results

As reported in the main text, recipients of AI-generated messages rated the author as less angry, smarter, more factual and logical, but less unique, and less likely to use narratives. Additionally, recipients of AI-generated messages, compared to human-generated messages, rated the author to be similarly assertive (b = -0.71, CI = [-3.45, 2.03], p = 0.612, BF01 = 35.53), moral (b = -0.01, CI = [-2.09, 2.08], p = 0.994, BF01 = 40.42), warm (b = -1.34, CI = [-3.18, 0.50], p = 0.154, BF01 = 14.59), authentic (b = -2.66, CI = [-5.56, 0.25], p = 0.073, BF01 = 6.29), creative (b = -1.06, CI = [-4.11, 1.99], p = 0.497, BF01 = 25.09), and interesting (b = -1.56, CI = [-4.66, 1.55], p = 0.326, BF01 = 19.49). The detailed results are summarized in Supplementary Table 5 below (also see osf.io/8yxvr for all supplementary tables).

Supplementary Table 5. Comparing Perceptions of the AI and Human Authors

		b		S.E.	*p*	95% CI		η^2 (in %)	β
Factual	(Intercept)	35.08	***	1.59	<.001	[31.96	38.20]		
	Pre-treatment DV	0.52	***	0.02	<.001	[0.48	0.56]	31.02	0.52
	AI conditoin	3.62	**	1.16	0.002	[1.34	5.90]	0.59	0.06
	Study	-19.20	***	1.20	<.001	[-21.54	-16.85]	13.61	-0.31
	F (3,1630) = 362.28, *R* -squared = 0.40, adjusted *R* -squared = 0.40								
Logical	(Intercept)	26.39	***	1.49	<.001	[23.46	29.32]		
	Pre-treatment DV	0.66	***	0.02	<.001	[0.62	0.69]	44.93	0.66
	AI condition	3.47	**	1.09	0.001	[1.33	5.61]	0.62	0.06
	Study	-6.53	***	1.12	<.001	[-8.74	-4.33]	2.03	-0.11
	F (3,1630) = 474.28, *R* -squared = 0.47, adjusted *R* -squared = 0.47								
Angry	(Intercept)	33.70	***	1.89	<.001	[30.00	37.40]		
	Pre-treatment DV	-0.02	ns	0.02	0.305	[-0.07	0.02]	0.06	-0.02
	AI condition	-7.25	***	1.38	<.001	[-9.95	-4.55]	1.67	-0.13
	Study	11.73	***	1.42	<.001	[8.95	14.52]	4.02	0.20
	F (3,1629) = 33.06, *R* -squared = 0.06, adjusted *R* -squared = 0.06								
Unique	(Intercept)	13.95	***	1.64	<.001	[10.73	17.17]		
	Pre-treatment DV	0.47	***	0.02	<.001	[0.43	0.52]	30.08	0.55
	AI conditoin	-3.91	**	1.45	0.007	[-6.75	-1.07]	0.73	-0.07
	F (2,997) = 222.29, *R* -squared = 0.31, adjusted *R* -squared = 0.31								
Vivid story-telling	(Intercept)	27.62	***	1.74	<.001	[24.20	31.04]		
	Pre-treatment DV	0.25	***	0.02	<.001	[0.20	0.30]	9.70	0.31
	AI conditoin	-9.44	***	1.54	<.001	[-12.46	-6.42]	3.64	-0.18
	F (2,996) = 76.47, *R* -squared = 0.13, adjusted *R* -squared = 0.13								
Assertive	(Intercept)	39.31	***	1.91	<.001	[35.55	43.06]		
	Pre-treatment DV	0.12	***	0.02	<.001	[0.07	0.16]	1.56	0.12
	AI conditoin	-0.71	ns	1.40	0.612	[-3.45	2.03]	0.02	-0.01
	Study	14.96	***	1.44	<.001	[12.13	17.79]	6.21	0.25
	F (3,1629) = 41.80, *R* -squared = 0.07, adjusted *R* -squared = 0.07								
Smart	(Intercept)	34.08	***	1.49	<.001	[31.17	36.99]		
	Pre-treatment DV	0.53	***	0.02	<.001	[0.49	0.56]	34.85	0.58
	AI conditoin	2.21	*	1.08	0.042	[0.08	4.34]	0.25	0.04
	Study	-9.86	***	1.12	<.001	[-12.05	-7.66]	4.55	-0.17
	F (3,1629) = 336.02, *R* -squared = 0.38, adjusted *R* -squared = 0.38								

Note . All models are linear regression models without adjustments. The Study variable is coded as 0 for Study 1, and 1 for Study 2. S.E. refers to standard error of the estimates. All results are two-tailed.

Supplementary Table 5 (continue). Comparing Perceptions of the AI and Human Authors

		b		S.E.	*p*	95% CI			η^2 (in %)	β
Moral	(Intercept)	27.34	***	1.46	<.001	[24.49	30.20	]		
	Pre-treatment DV	0.60	***	0.02	<.001	[0.56	0.63	]	41.52	0.65
	AI conditoin	-0.01	ns	1.06	0.994	[-2.09	2.08	]	0.00	0.00
	Study	0.21	ns	1.10	0.847	[-1.94	2.36	]	0.00	0.00
	F (3,1630) = 388.61, R-squared = 0.42, adjusted R-squared = 0.42									
Warm	(Intercept)	-16.27	***	1.29	<.001	[-18.79	-13.75	]		
	Pre-treatment DV	0.29	***	0.02	<.001	[0.26	0.32	]	18.02	0.26
	AI conditoin	-1.34	ns	0.94	0.154	[-3.18	0.50	]	0.12	-0.02
	Study	58.24	***	0.97	<.001	[56.35	60.14	]	68.98	0.82
	F (3,1629) = 1269.06, R-squared = 0.70, adjusted R-squared = 0.70									
Authentic	(Intercept)	29.44	***	1.67	<.001	[26.15	32.72	]		
	Pre-treatment DV	0.57	***	0.02	<.001	[0.53	0.62	]	37.67	0.61
	AI conditoin	-2.66	ns	1.48	0.073	[-5.56	0.25	]	0.32	-0.04
	F (2,997) = 306.6, R-squared = 0.38, adjusted R-squared = 0.38									
Creative	(Intercept)	19.83	***	1.76	<.001	[16.37	23.28	]		
	Pre-treatment DV	0.36	***	0.02	<.001	[0.31	0.40	]	17.43	0.42
	AI condition	-1.06	ns	1.55	0.497	[-4.11	1.99	]	0.05	-0.02
	F (2,997) = 106.42, R-squared = 0.18, adjusted R-squared = 0.17									
Interesting	(Intercept)	16.19	***	1.79	<.001	[12.67	19.71	]		
	Pre-treatment DV	0.60	***	0.03	<.001	[0.55	0.65	]	36.67	0.61
	AI condition	-1.56	ns	1.58	0.326	[-4.66	1.55	]	0.10	-0.02
	F (2,998) = 291.82, R-squared = 0.37, adjusted R-squared = 0.37									

Note. All models are linear regression models without adjustments. The Study variable is coded as 0 for Study 1, and 1 for Study 2. S.E. refers to standard error of the estimates. All results are two-tailed.

Analyses of the Linguistic Features of LLM and Human Messages

We also analyzed the linguistic features of the LLM and human messages separately for each study using Linguistic Inquiry and Word Count (LIWC) 2022. We report dictionary for selected LIWC dictionaries most used in prior work, and report for the full set of all dictionaries in Supplementary Table 6. The results in the main text report the aggregated results. All analyses were t-tests and all *p* values were corrected using Benjamini-Hochberg adjustment for multiple comparisons.

Supplementary Table 6. Linguistic Features of LLM and Human Messages

	Study 1				Study 2				Studies 1 and 2 Aggregated						
	AI	Human	*t*	*p*	AI	Human	*t*	*p*	AI	Human	*t*	*p*	*df*	95% CI	*d*
Analytic	87.40	55.55	8.53	0.000	78.25	62.16	4.95	0.000	82.82	58.85	9.48	0.000	198	[18.98 28.95]	1.34
Clout	49.83	52.22	-0.52	0.996	84.27	51.99	9.44	0.000	67.05	52.10	4.48	0.001	198	[8.36 21.53]	0.63
Authentic	9.12	32.64	-7.16	0.000	5.85	27.28	-6.65	0.000	7.48	29.96	-9.72	0.000	198	[-27.03 -17.91]	-1.38
Tone	32.41	23.76	1.71	0.996	2.44	4.43	-1.92	0.917	17.42	14.09	1.06	0.990	198	[-2.85 9.50]	0.15
WPS	18.58	20.17	-1.79	0.996	18.84	19.62	-0.89	0.917	18.71	19.90	-1.91	0.990	198	[-2.41 0.04]	-0.27
BigWords	26.92	21.21	5.84	0.000	24.25	21.79	3.02	0.221	25.58	21.50	6.31	0.000	198	[2.80 5.36]	0.89
Dic	81.02	86.33	-8.02	0.000	84.84	87.69	-4.03	0.009	82.93	87.01	-7.79	0.000	198	[-5.11 -3.05]	-1.10
Linguistic	58.66	67.25	-9.50	0.000	63.85	67.71	-4.78	0.001	61.26	67.48	-9.47	0.000	198	[-7.52 -4.93]	-1.34
function.	47.32	55.01	-8.11	0.000	51.75	55.53	-5.15	0.000	49.54	55.27	-9.00	0.000	198	[-6.98 -4.47]	-1.27
pronoun	5.09	10.97	-9.03	0.000	11.24	10.08	2.10	0.917	8.16	10.52	-4.46	0.001	198	[-3.40 -1.32]	-0.63
ppron	2.00	5.30	-6.90	0.000	5.71	5.19	1.26	0.917	3.85	5.25	-3.81	0.013	198	[-2.12 -0.67]	-0.54
i	0.00	0.48	-3.71	0.026	0.02	0.84	-5.25	0.000	0.01	0.66	-6.34	0.000	198	[-0.85 -0.45]	-0.90
we	1.07	1.22	-0.49	0.996	4.06	1.71	8.55	0.000	2.57	1.47	4.29	0.002	198	[0.59 1.60]	0.61
you	0.01	1.18	-4.18	0.005	0.00	0.42	-3.93	0.013	0.01	0.80	-5.16	0.000	198	[-1.09 -0.49]	-0.73
shehe	0.00	0.06	-1.24	0.996	0.00	0.04	-1.69	0.917	0.00	0.05	-1.87	0.990	198	[-0.10 0.00]	-0.27
they	0.57	1.76	-5.41	0.000	1.43	1.90	-1.90	0.917	1.00	1.83	-4.86	0.000	198	[-1.17 -0.49]	-0.69
ipron	3.09	5.66	-6.61	0.000	5.53	4.88	1.80	0.917	4.31	5.27	-3.28	0.080	198	[-1.54 -0.38]	-0.46
det	13.42	13.42	0.01	0.996	16.43	15.60	1.75	0.917	14.92	14.51	1.09	0.990	198	[-0.33 1.16]	0.15
article	7.39	6.10	3.35	0.081	7.74	7.26	1.48	0.917	7.57	6.68	3.44	0.050	198	[0.38 1.39]	0.49
number	1.75	0.92	3.68	0.028	0.53	1.16	-4.12	0.007	1.14	1.04	0.65	0.990	198	[-0.20 0.39]	0.09
prep	15.69	14.66	2.05	0.996	15.15	14.31	2.09	0.917	15.42	14.48	2.90	0.238	198	[0.30 1.58]	0.41
auxverb	8.77	9.60	-2.00	0.996	8.72	10.58	-4.79	0.001	8.75	10.09	-4.69	0.000	198	[-1.90 -0.78]	-0.66
adverb	2.94	5.38	-7.32	0.000	2.41	4.85	-7.64	0.000	2.68	5.11	-10.48	0.000	198	[-2.90 -1.98]	-1.48
conj	5.25	7.05	-4.93	0.000	5.12	6.05	-3.44	0.064	5.18	6.55	-5.89	0.000	198	[-1.82 -0.91]	-0.83
negate	0.73	2.01	-6.06	0.000	0.82	1.85	-7.02	0.000	0.78	1.93	-9.00	0.000	198	[-1.41 -0.90]	-1.27
verb	11.08	14.01	-5.46	0.000	13.85	16.91	-5.22	0.000	12.47	15.46	-6.75	0.000	198	[-3.87 -2.12]	-0.95
adj	9.15	8.73	0.92	0.996	6.54	7.22	-1.78	0.917	7.84	7.97	-0.39	0.990	198	[-0.78 0.52]	-0.06
quantity	6.03	4.28	4.19	0.005	4.12	4.72	-2.04	0.917	5.07	4.50	2.11	0.990	198	[0.04 1.11]	0.30
Drives	5.16	4.34	1.94	0.996	11.97	7.88	10.29	0.000	8.57	6.11	5.10	0.000	198	[1.51 3.40]	0.72
affiliation	1.95	1.75	0.53	0.996	5.76	2.21	10.28	0.000	3.86	1.98	5.89	0.000	198	[1.25 2.50]	0.83
achieve	2.01	0.82	6.98	0.000	0.87	1.11	-1.46	0.917	1.44	0.97	3.61	0.028	198	[0.21 0.73]	0.51
power	1.95	1.92	0.16	0.996	5.38	4.63	2.47	0.874	3.67	3.27	1.35	0.990	198	[-0.18 0.97]	0.19
Cognition	9.67	15.23	-8.39	0.000	9.98	15.33	-8.80	0.000	9.83	15.28	-12.19	0.000	198	[-6.33 -4.57]	-1.72
allnone	0.69	1.30	-2.89	0.304	0.74	1.34	-3.77	0.022	0.72	1.32	-4.60	0.001	198	[-0.86 -0.34]	-0.65
cogproc	8.75	13.91	-8.19	0.000	9.23	13.88	-7.85	0.000	8.99	13.89	-11.38	0.000	198	[-5.75 -4.05]	-1.61
insight	1.30	1.96	-2.87	0.323	0.80	2.07	-6.00	0.000	1.05	2.01	-6.13	0.000	198	[-1.27 -0.65]	-0.87
cause	2.62	2.57	0.21	0.996	2.78	2.60	0.77	0.917	2.70	2.58	0.69	0.990	198	[-0.22 0.46]	0.10
discrep	2.85	2.96	-0.36	0.996	2.83	2.94	-0.45	0.917	2.84	2.95	-0.56	0.990	198	[-0.50 0.28]	-0.08
tentat	0.37	2.29	-8.91	0.000	0.86	2.40	-7.59	0.000	0.62	2.34	-11.59	0.000	198	[-2.02 -1.43]	-1.64
certitude	0.06	0.46	-4.35	0.003	0.17	0.71	-4.92	0.000	0.12	0.58	-6.48	0.000	198	[-0.61 -0.32]	-0.92
differ	1.54	4.63	-7.87	0.000	1.70	4.02	-9.58	0.000	1.62	4.32	-11.68	0.000	198	[-3.17 -2.25]	-1.65
memory	0.00	0.01	-1.00	0.996	0.01	0.02	-0.41	0.917	0.01	0.02	-0.89	0.990	198	[-0.03 0.01]	-0.13

Note. All results are t-tests adjusted using Benjamini-Hochberg adjustment for multiple comparisons. All results are two-tailed.

Supplementary Table 6 (continue). Linguistic Features of LLM and Human Messages

	Study 1				Study 2				Studies 1 and 2 Aggregated							
	AI	Human	*t*	*p*	AI	Human	*t*	*p*	AI	Human	*t*	*p*	*df*	95% CI		*d*
Affect	4.65	4.33	1.08	0.996	9.86	7.67	5.72	0.000	7.25	6.00	3.18	0.110	198	[0.48	2.02]	0.45
tone_pos	2.54	1.97	1.96	0.996	2.92	2.03	4.03	0.009	2.73	2.00	3.99	0.007	198	[0.37	1.09]	0.56
tone_neg	2.09	2.33	-0.93	0.996	6.93	5.61	4.05	0.009	4.51	3.97	1.51	0.990	198	[-0.17	1.25]	0.21
emotion	0.67	1.04	-1.92	0.996	0.83	1.03	-1.46	0.917	0.75	1.03	-2.42	0.792	198	[-0.51	-0.05]	-0.34
emo_pos	0.22	0.38	-1.35	0.996	0.06	0.29	-4.20	0.005	0.14	0.34	-2.96	0.209	198	[-0.33	-0.07]	-0.42
emo_neg	0.25	0.58	-2.83	0.353	0.77	0.71	0.49	0.917	0.51	0.64	-1.55	0.990	198	[-0.31	0.04]	-0.22
emo_anx	0.03	0.03	-0.04	0.996	0.20	0.24	-0.57	0.917	0.12	0.14	-0.52	0.990	198	[-0.11	0.06]	-0.07
emo_anger	0.03	0.09	-1.40	0.996	0.31	0.21	1.54	0.917	0.17	0.15	0.53	0.990	198	[-0.06	0.11]	0.08
emo_sad	0.09	0.00	3.00	0.230	0.23	0.11	2.51	0.806	0.16	0.05	3.57	0.032	198	[0.05	0.17]	0.50
swear	0.00	0.00			0.00	0.01	-1.00	0.917	0.00	0.00	-1.00	0.990	198	[-0.01	0.00]	-0.14
Social	4.55	9.31	-7.91	0.000	13.69	11.11	4.41	0.002	9.12	10.21	-1.74	0.990	198	[-2.33	0.15]	-0.25
socbehav	1.43	1.93	-2.39	0.996	5.11	4.12	3.74	0.023	3.27	3.03	0.89	0.990	198	[-0.30	0.78]	0.13
prosocial	0.92	0.31	4.42	0.002	1.01	0.21	6.74	0.000	0.97	0.26	7.77	0.000	198	[0.53	0.89]	1.10
polite	0.01	0.06	-1.16	0.996	0.01	0.03	-0.84	0.917	0.01	0.04	-1.43	0.990	198	[-0.07	0.01]	-0.20
conflict	0.20	0.39	-1.73	0.996	3.18	2.58	2.86	0.342	1.69	1.49	0.93	0.990	198	[-0.23	0.64]	0.13
moral	0.03	0.34	-3.84	0.017	0.54	0.49	0.44	0.917	0.28	0.42	-1.83	0.990	198	[-0.28	0.01]	-0.26
comm	0.14	0.24	-1.39	0.996	0.12	0.54	-4.33	0.003	0.13	0.39	-4.23	0.003	198	[-0.38	-0.14]	-0.60
socrefs	2.95	7.09	-7.39	0.000	7.78	6.61	2.45	0.888	5.36	6.85	-3.38	0.061	198	[-2.35	-0.62]	-0.48
family	0.08	0.41	-2.86	0.323	0.25	0.35	-0.83	0.917	0.17	0.38	-2.53	0.626	198	[-0.39	-0.05]	-0.36
friend	0.00	0.02	-1.43	0.996	0.01	0.01	-0.29	0.917	0.00	0.01	-1.28	0.990	198	[-0.03	0.01]	-0.18
female	0.01	0.16	-2.02	0.996	0.00	0.06	-1.77	0.917	0.01	0.11	-2.59	0.543	198	[-0.19	-0.03]	-0.37
male	0.00	0.04	-1.42	0.996	0.00	0.12	-2.58	0.694	0.00	0.08	-2.92	0.228	198	[-0.13	-0.02]	-0.41
Culture	1.26	0.36	6.18	0.000	1.49	1.47	0.10	0.917	1.38	0.91	3.30	0.077	198	[0.19	0.74]	0.47
politic	1.24	0.27	7.04	0.000	1.47	1.32	0.72	0.917	1.36	0.79	4.12	0.004	198	[0.29	0.83]	0.58
ethnicity	0.02	0.03	-0.74	0.996	0.01	0.11	-2.47	0.874	0.01	0.07	-2.47	0.708	198	[-0.11	-0.01]	-0.35
tech	0.00	0.05	-1.31	0.996	0.01	0.04	-1.76	0.917	0.00	0.05	-1.91	0.990	198	[-0.09	0.00]	-0.27
Lifestyle	1.36	1.28	0.27	0.996	0.93	2.05	-5.03	0.000	1.15	1.67	-2.73	0.383	198	[-0.89	-0.14]	-0.39
leisure	0.09	0.19	-1.43	0.996	0.09	0.34	-2.90	0.304	0.09	0.27	-3.11	0.130	198	[-0.29	-0.06]	-0.44
home	0.04	0.09	-1.25	0.996	0.06	0.18	-2.59	0.689	0.05	0.13	-2.79	0.331	198	[-0.14	-0.02]	-0.39
work	0.82	0.73	0.40	0.996	0.52	1.08	-3.82	0.018	0.67	0.91	-1.86	0.990	198	[-0.50	0.01]	-0.26
money	0.55	0.39	0.89	0.996	0.29	0.50	-1.88	0.917	0.42	0.44	-0.22	0.990	198	[-0.24	0.19]	-0.03
relig	0.00	0.02	-1.00	0.996	0.01	0.02	-0.58	0.917	0.01	0.02	-1.11	0.990	198	[-0.04	0.01]	-0.16
Physical	7.20	5.45	3.30	0.092	1.87	2.30	-1.89	0.917	4.53	3.87	1.56	0.990	198	[-0.17	1.49]	0.22
health	4.46	2.83	4.28	0.004	0.51	0.43	0.50	0.917	2.49	1.63	2.77	0.346	198	[0.25	1.47]	0.39
illness	2.17	1.05	4.05	0.008	0.05	0.03	0.84	0.917	1.11	0.54	3.14	0.123	198	[0.21	0.93]	0.44
wellness	0.21	0.13	1.10	0.996	0.10	0.00	3.33	0.091	0.16	0.06	2.20	0.990	198	[0.01	0.17]	0.31
mental	0.00	0.06	-2.29	0.996	0.17	0.25	-0.76	0.917	0.08	0.15	-1.27	0.990	198	[-0.17	0.04]	-0.18
substances	1.29	1.20	0.42	0.996	0.00	0.00			0.65	0.60	0.32	0.990	198	[-0.23	0.32]	0.05
sexual	0.01	0.05	-1.17	0.996	0.00	0.02	-1.00	0.917	0.01	0.03	-1.52	0.990	198	[-0.06	0.01]	-0.22
food	0.03	0.18	-2.22	0.996	0.03	0.09	-1.38	0.917	0.03	0.13	-2.61	0.530	198	[-0.18	-0.02]	-0.37
death	1.03	0.30	5.65	0.000	1.02	1.08	-0.39	0.917	1.03	0.69	3.13	0.123	198	[0.13	0.55]	0.44
need	0.25	0.46	-1.68	0.996	0.84	0.90	-0.46	0.917	0.54	0.68	-1.39	0.990	198	[-0.34	0.06]	-0.20
want	0.03	0.33	-3.97	0.011	0.06	0.21	-3.14	0.161	0.05	0.27	-5.01	0.000	198	[-0.31	-0.13]	-0.71
acquire	0.09	0.47	-3.61	0.034	1.05	0.71	2.55	0.749	0.57	0.59	-0.26	0.990	198	[-0.22	0.17]	-0.04
lack	0.01	0.10	-1.82	0.996	0.04	0.14	-1.85	0.917	0.03	0.12	-2.60	0.535	198	[-0.16	-0.02]	-0.37
fulfill	0.01	0.04	-1.23	0.996	0.01	0.17	-3.15	0.155	0.01	0.10	-3.30	0.078	198	[-0.15	-0.04]	-0.47

Note. All results are t-tests adjusted using Benjamini-Hochberg adjustment for multiple comparisons. All results are two-tailed.

Supplementary Table 6 (continue). Linguistic Features of LLM and Human Messages

	Study 1				Study 2				Studies 1 and 2 Aggregated									
	AI	Human	*t*	*p*	AI	Human	*t*	*p*	AI	Human	*t*	*p*	*df*		95% CI			*d*
fatigue	0.00	0.00			0.00	0.01	-1.00	0.917	0.00	0.00	-1.00	0.990	198	[	-0.01	0.00	]	-0.14
reward	0.31	0.22	1.24	0.996	0.07	0.10	-0.74	0.917	0.19	0.16	0.71	0.990	198	[	-0.06	0.12	]	0.10
risk	3.28	1.45	7.16	0.000	2.60	1.30	6.20	0.000	2.94	1.38	9.31	0.000	198	[	1.24	1.90	]	1.32
curiosity	0.12	0.16	-0.67	0.996	0.22	0.11	1.90	0.917	0.17	0.14	0.78	0.990	198	[	-0.05	0.12	]	0.11
allure	3.10	5.86	-6.63	0.000	5.84	6.98	-2.42	0.917	4.47	6.42	-5.63	0.000	198	[	-2.63	-1.26	]	-0.80
Perception	8.84	9.17	-0.68	0.996	7.58	7.50	0.18	0.917	8.21	8.34	-0.37	0.990	198	[	-0.80	0.54	]	-0.05
attention	0.00	0.25	-3.20	0.127	0.55	0.20	3.32	0.093	0.27	0.23	0.67	0.990	198	[	-0.09	0.19	]	0.09
motion	0.35	0.60	-2.05	0.996	1.24	0.84	2.85	0.344	0.80	0.72	0.78	0.990	198	[	-0.12	0.29	]	0.11
space	8.40	7.75	1.42	0.996	5.73	6.12	-1.07	0.917	7.06	6.93	0.39	0.990	198	[	-0.52	0.78	]	0.06
visual	0.05	0.32	-3.39	0.071	0.18	0.37	-2.74	0.470	0.12	0.35	-4.34	0.002	198	[	-0.33	-0.13	]	-0.61
auditory	0.05	0.09	-0.77	0.996	0.02	0.07	-1.37	0.917	0.03	0.08	-1.41	0.990	198	[	-0.10	0.02	]	-0.20
feeling	0.01	0.18	-3.63	0.033	0.09	0.17	-1.96	0.917	0.05	0.18	-4.00	0.007	198	[	-0.19	-0.07	]	-0.57
time	0.94	2.39	-4.98	0.000	1.62	2.98	-5.53	0.000	1.28	2.69	-7.22	0.000	198	[	-1.79	-1.02	]	-1.02
focuspast	0.76	1.07	-1.51	0.996	1.40	2.44	-4.29	0.004	1.08	1.75	-3.84	0.012	198	[	-1.02	-0.33	]	-0.54
focuspresent	5.31	5.38	-0.20	0.996	5.22	5.47	-0.92	0.917	5.26	5.42	-0.73	0.990	198	[	-0.59	0.27	]	-0.10
focusfuture	0.32	1.46	-4.95	0.000	0.85	1.13	-1.52	0.917	0.58	1.29	-4.73	0.000	198	[	-1.01	-0.42	]	-0.67
Conversation	0.00	0.10	-2.97	0.250	0.01	0.12	-3.75	0.023	0.00	0.11	-4.75	0.000	198	[	-0.15	-0.06	]	-0.67
netspeak	0.00	0.03	-1.43	0.996	0.00	0.02	-1.41	0.917	0.00	0.02	-2.02	0.990	198	[	-0.05	0.00	]	-0.29
assent	0.00	0.06	-2.24	0.996	0.01	0.11	-3.53	0.048	0.00	0.08	-4.08	0.005	198	[	-0.12	-0.04	]	-0.58
nonflu	0.00	0.01	-1.00	0.996	0.00	0.00			0.00	0.01	-1.00	0.990	198	[	-0.02	0.01	]	-0.14
filler	0.00	0.00			0.00	0.00			0.00	0.00								
AllPunc	12.62	11.18	2.11	0.996	9.74	11.17	-2.68	0.545	11.18	11.17	0.01	0.990	198	[	-0.89	0.90	]	0.00
Period	5.54	5.25	1.14	0.996	5.37	5.57	-0.75	0.917	5.45	5.41	0.23	0.990	198	[	-0.32	0.40	]	0.03
Comma	5.48	3.60	4.77	0.001	3.26	3.41	-0.49	0.917	4.37	3.50	3.16	0.118	198	[	0.32	1.40	]	0.45
QMark	0.00	0.17	-2.69	0.515	0.00	0.23	-2.92	0.293	0.00	0.20	-3.98	0.007	198	[	-0.30	-0.10	]	-0.56
Exclam	0.00	0.03	-1.42	0.996	0.00	0.03	-2.05	0.917	0.00	0.03	-2.42	0.792	198	[	-0.06	-0.01	]	-0.34
Apostro	0.29	1.01	-4.18	0.005	0.31	0.85	-3.11	0.169	0.30	0.93	-5.17	0.000	198	[	-0.87	-0.39	]	-0.73
OtherP	1.32	1.12	0.68	0.996	0.81	1.08	-1.18	0.917	1.06	1.10	-0.21	0.990	198	[	-0.40	0.33	]	-0.03

Note. All results are t-tests adjusted using Benjamini-Hochberg adjustment for multiple comparisons. All results are two-tailed.

Moderators of the Persuasion Effects

We probed the moderating role of two variables, party identity and pre-treatment policy support level, on the persuasive effect of AI-generated messages, human-generated messages, and AI-generated messages selected by humans (Human-in-the-Loop).

Analysis Strategy

We combined the data from Studies 1 and 2. Like the main analysis reported in the main text, the post-treatment measure of the dependent variable was regressed on the dummy-coded variables for Human-in-the-Loop, LLM, and Human conditions (all contrasted with the neutral Control condition), while controlling for pre-treatment policy support. We also included a dummy variable for Study. To test pre-treatment policy support as a moderator, we added interaction terms between pre-treatment policy support and each of the treatment condition dummy variables. To test party identity as a moderator, we added a term for party identity (seven-point scale recorded to run from 0 = strong Democrat to 1 = strong Republican at the increment of ⅙; "Party ID"), and interaction terms between party identity and each of the treatment conditions. In the model testing party identity as a moderator, we still controlled for pre-treatment policy support.

Results

We found evidence that party identity moderates the effects of the messages. The Party ID × LLM condition (b = -5.16, CI = [-8.16, -2.16], $p < .001$) and Party ID × Human condition (b = -3.93, CI = [-6.95, -0.90], p = 0.011) were both negative and significant, suggesting that participants who self-reported as Republican were less persuaded by the AI-generated and human-generated messages. The Human-in-the-Loop condition × Party ID interaction effect was not significant (b = -2.42, CI = [-5.48, 0.65], p = 0.122).

We also found evidence that pre-treatment policy support moderates the effect of AI-generated messages. The pre-treatment policy support × LLM condition interaction effect was significant (b = 0.05, CI = [0.02, 0.08], p = 0.001), suggesting that participants with higher pre-treatment support were more persuaded by the AI-generated messages. The pre-treatment policy support × Human condition (b = 0.00, CI = [-0.03, 0.03], p = 0.988) and the pre-treatment policy support × Human-in-the-Loop condition interaction effects were not significant (b = 0.01, CI = [-0.02, 0.04], p = 0.468). The detailed results of the two models can be found in Supplementary Table 7 below (also see osf.io/8yxvr for all supplementary tables).

Supplementary Table 7. Studies 1 and 2 Results: Moderating Effects of Party Identity and Pre-Treatment Support

		b	S.E	*p*	95% CI		η^2 (in %)	β
Model 1	(Intercept)	0.14	0.76	0.852	[-1.35	1.64]		
	Pre-Treatment DV	1.02	0.01	<.001	[1.01	1.03]	89.92	0.94
	Human-in-the-Loop condition	4.41	0.86	<.001	[2.73	6.09]	1.42	0.06
	Party ID	-0.98	1.10	0.373	[-3.15	1.18]	1.20	-0.01
	AI condition	4.77	0.84	<.001	[3.12	6.42]	0.79	0.06
	Human condition	4.51	0.84	<.001	[2.87	6.15]	1.02	0.06
	Study	-0.82	0.36	0.024	[-1.54	-0.11]	0.16	-0.01
	Human-in-the-Loop condition × Party ID	-2.42	1.56	0.122	[-5.48	0.65]	0.07	-0.02
	Party ID × AI condition	-5.16	1.53	<.001	[-8.16	-2.16]	0.35	-0.04
	Party ID × Human condition	-3.93	1.54	0.011	[-6.95	-0.90]	0.20	-0.03
	$F(9,3209) = 3952.33$, R-squared = 0.92, adjusted R-squared = 0.92							
Model 2	(Intercept)	0.01	0.72	0.986	[-1.39	1.42]		
	Pre-Treatment DV	1.02	0.01	<.001	[1.00	1.04]	91.47	0.94
	Human-in-the-Loop condition	2.77	0.97	0.004	[0.86	4.67]	1.45	0.03
	AI condition	-0.30	0.98	0.757	[-2.22	1.62]	0.79	0.00
	Human condition	2.85	0.98	0.004	[0.93	4.77]	1.08	0.04
	Study	-1.25	0.36	<.001	[-1.96	-0.55]	0.38	-0.02
	Pre-Treatment DV × Human-in-the-Loop condition	0.01	0.02	0.468	[-0.02	0.04]	0.02	0.01
	Pre-Treatment DV × AI condition	0.05	0.02	0.001	[0.02	0.08]	0.32	0.04
	Pre-Treatment DV × Human condition	0.00	0.02	0.988	[-0.03	0.03]	0.00	0.00
	$F(8,3210) = 4390.81$, R-squared = 0.92, adjusted R-squared = 0.92							

Note. All models are linear regression models without adjustments. The Study variable is coded as 0 for Study 1, and 1 for Study 2. The model's reference group is the Control condition. S.E. refers to standard error of the estimates. All results are two-tailed.

Mediators of the Persuasion Effects

We conducted a mediation analysis to explore how different attributes of the AI and human messages may contribute to their persuasiveness relative to each other. We are unable to conduct mediation analyses of what drives the persuasive effect of AI messages relative to the neutral messages in the Control condition. This is because participants' responses to the questions about the features of the neutral control messages - which are on topics such as skiing and neckties - are not comparable to participants' responses in the Human or LLM conditions.

Despite the lack of a significant total effect of message authorship on persuasiveness, indirect effects revealed from mediation analyses can offer nuanced insights into the underlying process regarding which pathways AI and humans each attain a similar level of persuasiveness.

We conducted 5 mediation analyses only using responses from participants in the LLM and Human conditions in Studies 1 and 2. We assessed the mediating roles of the five attributes that we identified AI and humans differ (being informed, logical, angry, unique, and using vivid-story telling) on the score of policy change support (post-treatment support level - pre-treatment support level).

In each model, the condition indicator (1 = AI, 0 = Human) predicted one of the five mediators: informed (a1), logical (a2), angry (a3), unique (a4), and vivid-story telling (a5) not simultaneously. These mediators in turn predicted the change score (b1-b5). The corresponding indirect effects from the condition variable to the dependent variables via each of the five pathways were estimated and 95% confidence intervals were estimated based on 5000 bootstrap samples. For the models about informed, logical, and angry, the fixed effects of Study were also added, given that these measures were asked in both studies.

The five mediation models are summarized in Supplementary Table 8a, and each row of the table has results for a model with a different mediator. Reviewing the indirect effects, the indirect effects of all five mediators are significant, as their 95% confidence intervals do not overlap with 0. The AI author was perceived as being more informed ($b = 3.40$, $p = .015$, CI = [0.66, 6.15]), logical ($b = 3.19$, $p = .030$, CI = [0.31, 6.07]), and less angry ($b = -7.24$, $p < .001$, CI = [-9.94, -4.54]), while the human authors were perceived as being more unique ($b = -5.72$, $p = .001$, CI = [-9.11, -2.33]) and more likely to use vivid storytelling ($b = -10.41$, $p < .001$, CI = [-13.58, -7.24]). Perceptions of being more informed ($b = 0.12$, $p < .001$, CI = [0.10, 0.14]), logical ($b = 0.12$, $p < .001$, CI = [0.10, 0.14]), unique ($b = 0.10$, $p < .001$, CI = [0.08, 0.13]), using vivid storytelling ($b = 0.07$, $p < .001$, CI = [0.05, 0.09]), and being less angry ($b = -0.04$, $p < .001$, CI = [-0.06, -0.02]) are associated with a greater level of persuasion effect.

Supplementary Table 8a. Mediation Analysis (Mediators Tested Separately)

Mediator	Path a	p	Path b	p	Path c (Total)	p	Path c' (Direct)	p	Indirect Effect [95%CI]
Informed (1)	3.40 (1.40)	0.015	0.12 (0.01)	<.001	-0.36 (0.53)	0.497	-0.76 (0.50)	0.13	0.40 [0.07 0.74]
Logical (2)	3.19 (1.47)	0.03	0.12 (0.01)	<.001	-0.36 (0.53)	0.497	-0.74 (0.50)	0.138	0.38 [0.03 0.72]
Angry (3)	-7.24 (1.38)	<.001	-0.04 (0.01)	<.001	-0.35 (0.53)	0.503	-0.63 (0.53)	0.234	0.28 [0.12 0.47]
Unique (4)	-5.72 (1.73)	0.001	0.10 (0.01)	<.001	-0.72 (0.64)	0.257	-0.13 (0.61)	0.839	-0.60 [-1.01 -0.24]
Vivid Storytelling (5)	-10.41 (1.62)	<.001	0.07 (0.01)	<.001	-0.74 (0.64)	0.242	-0.02 (0.64)	0.975	-0.72 [-1.21 -0.35]

Note. All models are mediation models without adjustments. The effects are reported in the format of coefficient (standard error). All results are two-tailed.

These mediation analysis findings suggest that, while AI and human messages attain similar levels of persuasiveness, they do so through at least somewhat distinct pathways: the persuasiveness of AI-generated messages (relative to human-generated messages) is driven in part by its ability to use evidence (indirect effect = 0.40, CI = [0.07, 0.75]), logical reasoning (indirect effect = 0.38, CI = [0.04, 0.74]), and a dispassionate voice (less "angry"; indirect effect = 0.28, CI = [0.12, 0.48]). By contrast, the persuasiveness of human-generated messages (relative to AI-generated messages) is driven in part by the perceived uniqueness (indirect effect = -0.60, CI = [-1.00, -0.23]) and originality (indirect effect = -0.72, CI = [-1.18, -0.36]) of the messages. This nuanced understanding of AI and human communication styles not only enriches our results but also offers valuable insights into effective message crafting in different contexts.

These results should also be interpreted with caution, as mediation models are inherently limited by their ability to disentangle the effects of multiple potential pathways. Specifically, we also estimated a model with all 5 mediators entered into the model simultaneously, as summarized in Supplementary Table 8b. The anger pathway is the only significant indirect effect (b = 0.24, CI = [0.06, 0.48]). However, the lack of the remaining pathways' effects need not indicate that they play no role in explaining the persuasion effects. This is because, in a mediation model with multiple parallel mediators, one mediator's significance (while others are not) could indicate either (a) the significant mediator being the "real" or primary mediator, or (b) the significant mediator being the last in a causal chain involving multiple mediators. Therefore, the lack of indirect effects in the pathways involving the messages' being informed, logical, unique, or using vivid story-telling may be a result of their effects being further mediated by the anger pathway.

Supplementary Table 8b. Mediation Analysis (Mediators Tested Simultaneously)

Mediator	Path a	p	Path b	p	Path c (Total)	p	Path c' (Direct)	p	Indirect Effect [95%CI]
Informed (1)	-1.45 (1.86)	0.437	0.04 (0.02)	0.027					-0.06 [-0.24 0.09]
Logical (2)	0.34 (2.00)	0.866	0.06 (0.02)	0.002					0.02 [-0.22 0.27]
Angry (3)	-7.19 (1.81)	<.001	-0.03 (0.01)	0.002	-0.74 (0.64)	0.242	-0.73 (0.61)	0.232	0.24 [0.06 0.49]
Unique (4)	-5.78 (1.73)	0.001	0.03 (0.02)	0.127					-0.16 [-0.41 0.05]
Vivid Storytelling (5)	-10.41 (1.62)	<.001	0.01 (0.02)	0.693					-0.06 [-0.45 0.29]

Note. All models are mediation models without adjustments. The effects are reported in the format of coefficient (standard error). All results are two-tailed.

We also considered testing other variables as mediators, including the policy evaluation measures (e.g., the policy is a "smart idea") and objective text features (Linguistic Inquiry and Word Count, or LIWC). However, as reported in the main text, none of the policy evaluation

measures differ across the LLM and Human conditions, disqualifying them as potential mediators. For objective text features, any potential mediation analyses would occur on the message-level as opposed to individual-level. Due to the small number of messages and the brevity of these messages, we were not able to conduct mediation analyses with sufficient power.

Correlations Between Message Features and Message Persuasiveness

We also analyzed how ratings of authors, policies, and text features (Linguistic Inquiry and Word Count, or LIWC) were correlated with the messages' persuasiveness (the score of policy change support measured by the post-treatment support level - pre-treatment support level). We did so separately for the messages generated by the LLM and humans. These results are summarized in Supplementary Tables 9 and 10. Supplementary Table 9 summarizes the correlations between participants' ratings of authors and policies and the messages' persuasiveness, so the analyses were conducted at the level of participants. Supplementary Table 10 summarizes the correlations between the messages' text features derived from LIWC and the messages' persuasiveness, so the analyses were conducted at the level of the message. For both sets of analyses, the data across Studies 1 and 2 were combined. For the results summarized in Supplementary Table 10, the *p* values were corrected using Hochberg adjustment.

Reviewing these results, there are few differences in the correlations across the LLM and human messages. These are within our expectations, certain features may be consistently influential in persuasion, independent of whether the message is LLM-created or human-written. We also note that participants do not know the authors are sometimes LLM.

Supplementary Table 9. Correlation between Perceived Features of the Messages (at Participant Level) in Each Condition and Change in Support

		Human	*p*	AI	*p*
Rating of the Author	Informed	0.29	<.001	0.34	<.001
	Logical	0.33	<.001	0.35	<.001
	Angry	-0.11	0.001	-0.11	0.002
	Unique	0.24	<.001	0.32	<.001
	Vivid story-telling	0.18	<.001	0.18	<.001
	Smart	0.32	<.001	0.33	<.001
	Moral	0.32	<.001	0.35	<.001
	Warm	0.29	<.001	0.28	<.001
	Authentic	0.25	<.001	0.34	<.001
	Assertive	-0.03	0.448	-0.02	0.661
Rating of the Policy	Smart	0.35	<.001	0.45	<.001
	Compassionate	0.32	<.001	0.35	<.001
	Ethical	0.33	<.001	0.39	<.001

Note. All results are correlations adjusted using Benjamini-Hochberg adjustment for multiple comparisons. All results are two-tailed.

Supplementary Table 10. Correlation between Features of the Messages (at Message Level) in Each Condition and Change in Support

	Human	*p*	AI		*p*
Analytic	0.14	0.996	0.30		0.191
Clout	-0.02	0.996	-0.29		0.299
Authentic	0.06	0.996	0.29		0.328
Tone	-0.01	0.996	0.15		1.000
WPS	0.02	0.996	0.00		1.000
BigWords	0.16	0.996	0.19		1.000
Dic	-0.2	0.996	-0.26		0.746
Linguistic	-0.23	0.996	-0.37	*	0.014
function.	-0.26	0.996	-0.37	*	0.015
pronoun	-0.15	0.996	-0.37	*	0.014
ppron	-0.2	0.996	-0.34		0.062
i	-0.17	0.996	-0.10		1.000
we	-0.14	0.996	-0.29		0.249
you	0	0.996	0.15		1.000
shehe	0.11	0.996			
they	-0.16	0.996	-0.30		0.186
ipron	0.02	0.996	-0.34		0.063
det	0.02	0.996	-0.26		0.606
article	-0.03	0.996	0.01		1.000
number	0.13	0.996	0.26		0.714
prep	-0.01	0.996	0.20		1.000
auxverb	-0.17	0.996	-0.37	*	0.019
adverb	-0.05	0.996	0.21		1.000
conj	-0.11	0.996	0.06		1.000
negate	-0.02	0.996	-0.10		1.000
verb	-0.17	0.996	-0.44	***	0.000

Note . All results are correlations adjusted using Benjamini-Hochberg adjustment for multiple comparisons. All results are two-tailed.

Supplementary Table 10 (continue). Correlation between Features of the Messages (at Message Level) in Each Condition and Change in Support

	Human	*p*	AI		*p*
adj	0.08	0.996	0.04		1.000
quantity	0.05	0.996	0.12		1.000
Drives	-0.23	0.996	-0.36	*	0.027
affiliation	-0.06	0.996	-0.33		0.067
achieve	-0.17	0.996	0.05		1.000
power	-0.18	0.996	-0.26		0.623
Cognition	-0.12	0.996	-0.19		1.000
allnone	0.05	0.996	0.10		1.000
cogproc	-0.14	0.996	-0.27		0.559
insight	0.03	0.996	0.12		1.000
cause	-0.01	0.996	-0.07		1.000
discrep	-0.22	0.996	-0.32		0.109
tentat	-0.07	0.996	-0.24		1.000
certitude	-0.07	0.996	-0.12		1.000
differ	-0.14	0.996	-0.04		1.000
memory	-0.03	0.996	-0.10		1.000
Affect	-0.15	0.996	-0.33		0.072
tone_pos	-0.05	0.996	-0.13		1.000
tone_neg	-0.13	0.996	-0.30		0.189
emotion	-0.02	0.996	-0.12		1.000
emo_pos	-0.01	0.996	0.02		1.000
emo_neg	-0.04	0.996	-0.22		1.000
emo_anx	0	0.996	-0.11		1.000
emo_anger	-0.03	0.996	-0.18		1.000
emo_sad	0.01	0.996	-0.18		1.000
swear	-0.1	0.996			
Social	-0.15	0.996	-0.33		0.089
socbehav	-0.08	0.996	-0.30		0.204

Note. All results are correlations adjusted using Benjamini-Hochberg adjustment for multiple comparisons. All results are two-tailed.

Supplementary Table 10 (continue). Correlation between Features of the Messages (at Message Level) in Each Condition and Change in Support

	Human		*p*	AI		*p*
prosocial	-0.05		0.996	-0.07		1.000
polite	0.09		0.996	-0.01		1.000
conflict	-0.09		0.996	-0.28		0.343
moral	0.17		0.996	-0.25		0.973
comm	-0.07		0.996	0.08		1.000
socrefs	-0.13		0.996	-0.30		0.215
family	0.08		0.996	-0.08		1.000
friend	0.19		0.996	-0.04		1.000
female	0.15		0.996	0.06		1.000
male	-0.04		0.996			
Culture	0.03		0.996	0.12		1.000
politic	-0.01		0.996	0.10		1.000
ethnicity	0.04		0.996	0.15		1.000
tech	0.16		0.996	-0.01		1.000
Lifestyle	0		0.996	0.01		1.000
leisure	-0.01		0.996	-0.04		1.000
home	-0.03		0.996	-0.24		1.000
work	0.05		0.996	0.18		1.000
money	-0.08		0.996	-0.09		1.000
relig	0.35	*	0.042	-0.02		1.000
Physical	0.11		0.996	0.41	**	0.003
health	0.01		0.996	0.39	**	0.005
illness	0.13		0.996	0.43	***	0.001
wellness	-0.11		0.996	-0.02		1.000
mental	-0.07		0.996	-0.07		1.000
substances	0.04		0.996	0.08		1.000
sexual	0.02		0.996	0.06		1.000
food	0.04		0.996	0.00		1.000

Note. All results are correlations adjusted using Benjamini-Hochberg adjustment for multiple comparisons. All results are two-tailed.

Supplementary Table 10 (continue). Correlation between Features of the Messages (at Message Level) in Each Condition and Change in Support

	Human		*p*	AI		*p*
death	-0.04		0.996	0.19		1.000
need	-0.13		0.996	-0.30		0.229
want	-0.03		0.996	-0.06		1.000
acquire	0		0.996	-0.25		0.766
lack	-0.05		0.996	-0.06		1.000
fulfill	0.14		0.996	-0.04		1.000
fatigue	-0.06		0.996			
reward	0.12		0.996	-0.06		1.000
risk	0.02		0.996	0.14		1.000
curiosity	0.13		0.996	-0.01		1.000
allure	-0.01		0.996	-0.27		0.559
Perception	0.13		0.996	0.20		1.000
attention	-0.02		0.996	-0.28		0.338
motion	-0.14		0.996	-0.23		1.000
space	0.22		0.996	0.32		0.099
visual	-0.12		0.996	-0.04		1.000
auditory	-0.04		0.996	0.10		1.000
feeling	0.13		0.996	-0.09		1.000
time	0		0.996	-0.07		1.000
focuspast	-0.01		0.996	-0.06		1.000
focuspresent	-0.09		0.996	0.14		1.000
focusfuture	0.01		0.996	-0.37	*	0.016
Conversation	-0.07		0.996	0.01		1.000
netspeak	0.08		0.996			
assent	-0.15		0.996	0.01		1.000
nonflu	0.1		0.996			
filler						
AllPunc	0		0.996	0.31		0.168
Period	0		0.996	-0.01		1.000
Comma	-0.07		0.996	0.25		0.888
QMark	-0.09		0.996			
Exclam	0.22		0.996			
Apostro	-0.09		0.996	0.15		1.000
OtherP	0.15		0.996	0.24		1.000

Note. All results are correlations adjusted using Benjamini-Hochberg adjustment for multiple comparisons. All results are two-tailed.

Comparison of GPT 3 and GPT 4 Messages

Since the conclusion of our research, more powerful LLM models have been developed and become accessible to the public. For example, our research used GPT 3 and GPT 3.5 which were available at the time of our research in late 2022, whereas GPT 4 is widely available at the time of preparation of the below analyses of this document in late 2023. To probe into the question of whether our results are dependent on LLM models, we compared the 50 messages generated from Study 1 using GPT 3 on October 26, and 50 messages generated using GPT 4 ("ChatGPT") on November 8 2023 using the same prompt. We found there is a great deal of linguistic similarity between these LLMs. As such, it is likely that the effects we reported in the main text would also be observed if messages generated by more recent models, such as GPT 4, were tested.

Specifically, we compared their features in Linguistic Inquiry and Word Count (LIWC) 2022 and summarized the results in Supplementary Table 11. Most features are similar across the messages from the two models. For example, they use a similar number of words reflecting "Analytic Thinking" and first-person pronouns (ps > .900). However, some differences still emerged from the comparisons. For example, GPT 4, on average, uses words that are longer ($p < .001$), and has more words per message (GPT 3 mean = 188.94, GPT 4 mean = 253.88). Additionally, GPT 4 use higher grade-level vocabulary than GPT 3 (GPT 3 mean = 10.57, GPT 4 mean = 12.42, $p < .001$). All analyses were t-tests and all p values were corrected using Benjamini-Hochberg adjustment for multiple comparisons.

Supplementary Table 11. Comparison of the Linguistic Features of GPT 3 and GPT4

	GPT 3.5	GPT 4		*p*
Analytic	87.40	87.01		0.973
Clout	49.83	59.52		0.176
Authentic	9.12	18.38	***	0.000
Tone	32.41	47.53		0.110
WPS	18.58	19.31		0.973
BigWords	26.92	30.25	**	0.001
Dic	81.02	83.38	**	0.006
Linguistic	58.66	60.20		0.973
function.	47.32	47.38		0.973
pronoun	5.09	7.35	***	0.000
ppron	2.00	2.95	*	0.043
i	0.00	0.01		0.973
we	1.07	2.06	**	0.009
you	0.01	0.09		0.330
shehe	0.00	0.00		
they	0.57	0.55		0.973
ipron	3.09	4.40	***	0.000
det	13.42	15.65	***	0.000
article	7.39	9.92	***	0.000
number	1.75	0.74	***	0.000
prep	15.69	13.72	***	0.000
auxverb	8.77	6.54	***	0.000
adverb	2.94	3.95	*	0.013
conj	5.25	5.13		0.973
negate	0.73	1.61	***	0.000
verb	11.08	9.05	***	0.000
adj	9.15	10.70	***	0.000
quantity	6.03	4.00	***	0.000
Drives	5.16	6.51	*	0.028
affiliation	1.95	3.31	**	0.002
achieve	2.01	1.26	**	0.002
power	1.95	2.31		0.973
Cognition	9.67	10.39		0.973

Note. All results are t-tests adjusted using Benjamini-Hochberg adjustment for multiple comparisons. All results are two-tailed.

Supplementary Table 11 (continue). Comparison of the Linguistic Features of GPT 3 and GPT4

	GPT 3.5	GPT 4		*p*
allnone	0.69	1.33	**	0.001
cogproc	8.75	8.99		0.973
insight	1.30	1.77		0.110
cause	2.62	1.74	***	0.000
discrep	2.85	1.82	**	0.007
tentat	0.37	0.43		0.973
certitude	0.06	0.42	***	0.000
differ	1.54	2.88	***	0.000
memory	0.00	0.02		0.973
Affect	4.65	5.46		0.054
tone_pos	2.54	3.61	**	0.002
tone_neg	2.09	1.85		0.973
emotion	0.67	0.99		0.973
emo_pos	0.22	0.58	*	0.013
emo_neg	0.25	0.26		0.973
emo_anx	0.03	0.03		0.973
emo_anger	0.03	0.07		0.973
emo_sad	0.09	0.03		0.973
swear	0.00	0.00		
Social	4.55	9.19	***	0.000
socbehav	1.43	2.86	***	0.000
prosocial	0.92	1.15		0.973
polite	0.01	0.11		0.163
conflict	0.20	0.29		0.973
moral	0.03	0.19		0.076
comm	0.14	0.31		0.325
socrefs	2.95	5.80	***	0.000
family	0.08	0.18		0.973
friend	0.00	0.00		
female	0.01	0.04		0.973
male	0.00	0.02		0.973
Culture	1.26	0.35	***	0.000

Note. All results are t-tests adjusted using Benjamini-Hochberg adjustment for multiple comparisons. All results are two-tailed.

Supplementary Table 11 (continue). Comparison of the Linguistic Features of GPT 3 and GPT4

	GPT 3.5	GPT 4		*p*
politic	1.24	0.35	***	0.000
ethnicity	0.02	0.00		0.973
tech	0.00	0.00		
Lifestyle	1.36	1.01		0.973
leisure	0.09	0.17		0.973
home	0.04	0.01		0.973
work	0.82	0.56		0.973
money	0.55	0.28		0.973
relig	0.00	0.02		0.973
Physical	7.20	7.18		0.973
health	4.46	5.54		0.144
illness	2.17	1.35		0.160
wellness	0.21	0.46		0.160
mental	0.00	0.03		0.973
substances	1.29	0.94		0.973
sexual	0.01	0.04		0.973
food	0.03	0.10		0.973
death	1.03	0.10	***	0.000
need	0.25	0.68	**	0.005
want	0.03	0.05		0.973
acquire	0.09	0.25		0.447
lack	0.01	0.01		0.973
fulfill	0.01	0.02		0.973
fatigue	0.00	0.01		0.973
reward	0.31	0.29		0.973
risk	3.28	1.78	***	0.000
curiosity	0.12	0.17		0.973
allure	3.10	2.69		0.973
Perception	8.84	8.94		0.973
attention	0.00	0.21	***	0.000
motion	0.35	0.70	**	0.008
space	8.40	7.82		0.973
visual	0.05	0.20		0.111
auditory	0.05	0.05		0.973

Note. All results are t-tests adjusted using Benjamini-Hochberg adjustment for multiple comparisons. All results are two-tailed.

Supplementary Table 11 (continue). Comparison of the Linguistic Features of GPT 3 and GPT4

	GPT 3.5	GPT 4		*p*
feeling	0.01	0.02		0.973
time	0.94	1.48	**	0.007
focuspast	0.76	0.41		0.434
focuspresent	5.31	5.25		0.973
focusfuture	0.32	0.42		0.973
Conversation	0.00	0.01		0.973
netspeak	0.00	0.00		
assent	0.00	0.01		0.973
nonflu	0.00	0.00		
filler	0.00	0.00		
AllPunc	12.62	15.42	***	0.000
Period	5.54	5.28		0.973
Comma	5.48	7.04	***	0.000
QMark	0.00	0.02		0.973
Exclam	0.00	0.00		
Apostro	0.29	1.29	***	0.000
OtherP	1.32	1.80		0.716

Note. All results are t-tests adjusted using Benjamini-Hochberg adjustment for multiple comparisons. All results are two-tailed.

Author Identity Tabulation

We examined whether participants perceived the messages to be generated by LLM or humans.

Measure

Participants in Study 2 were asked the question “The message is most likely written by which of the following?” and given the choice of: (a) “An adult person”, (b) “A group of people”, (c) “An expert on the topic”, (d) “An artificial intelligence program”, (e) “An intelligent adolescent”, (f) “An elementary school-age child”, and (g) “Other (please specify)”. We coded answers a, b, c, e, and f as human. After reviewing the open-ended answers for participants who chose g, we coded those answers as human as well. We coded answer d as AI.

Results

Most participants in both the LLM condition and the Human condition perceived the author to be human. In the LLM condition, 94.4% of participants answered that the messages were generated by humans. In the Human condition, 94.7% of participants answered that the messages were generated by humans.

Participants’ Change in Policy Support by Condition Across Studies

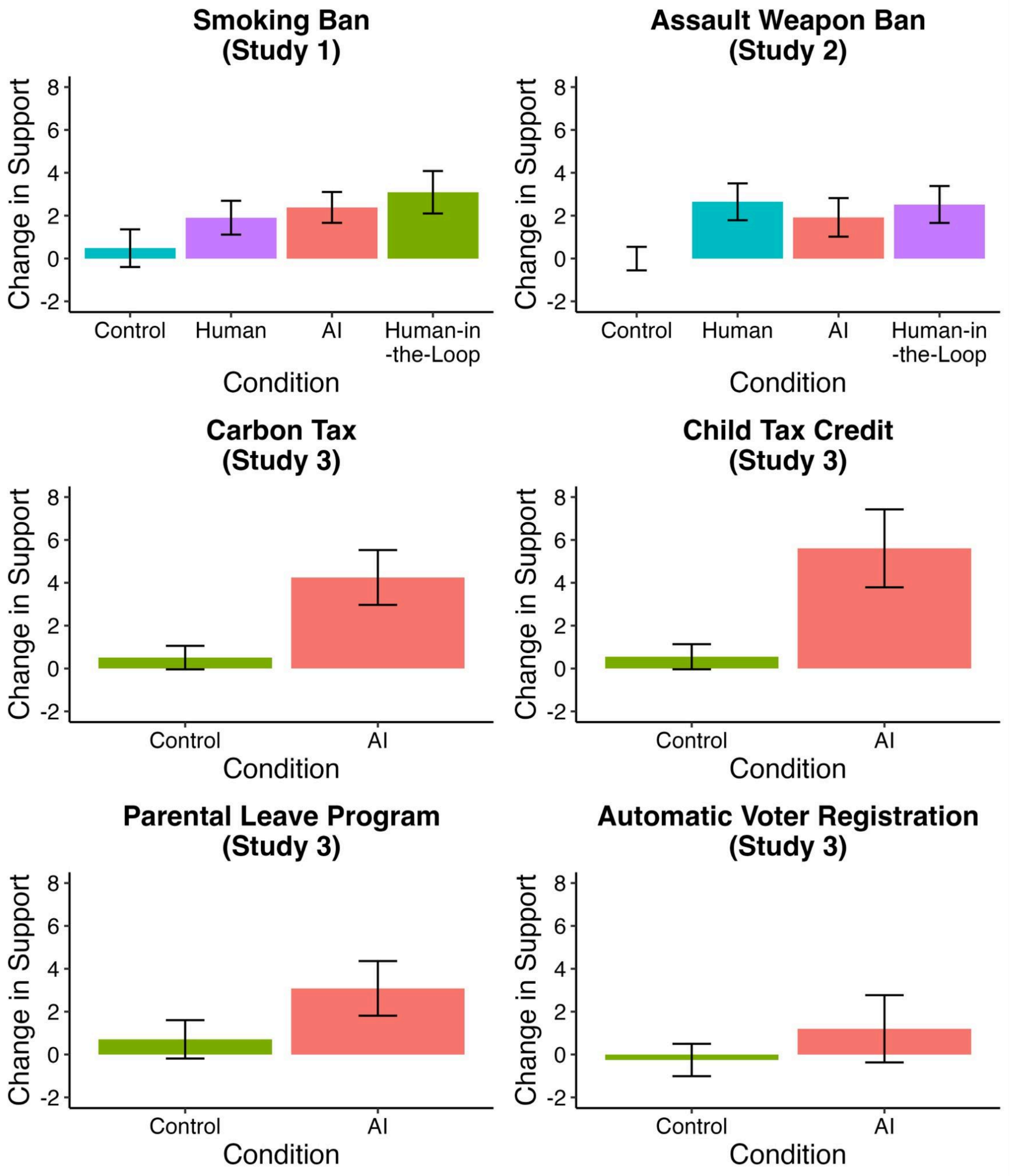

Supplementary Figure 1. Participants’ Change in Policy Support by Condition Across Studies. *Note. Y-axes represent the average difference between participants’ post-treatment and pre-treatment policy support (both scaled from 0 to 100, 100 = highest level of support). Data are presented as mean values with 95% confidence intervals. Higher scores indicate participants became more supportive of the policy.* $N_{Study\ 1}$ *= 1,203 participants,* $N_{Study\ 2}$ *= 2,016 participants,* $N_{Study\ 3}$ *= 1,610 participants.*

Different Perceptions of Human and AI Authors in Studies 1 and 2

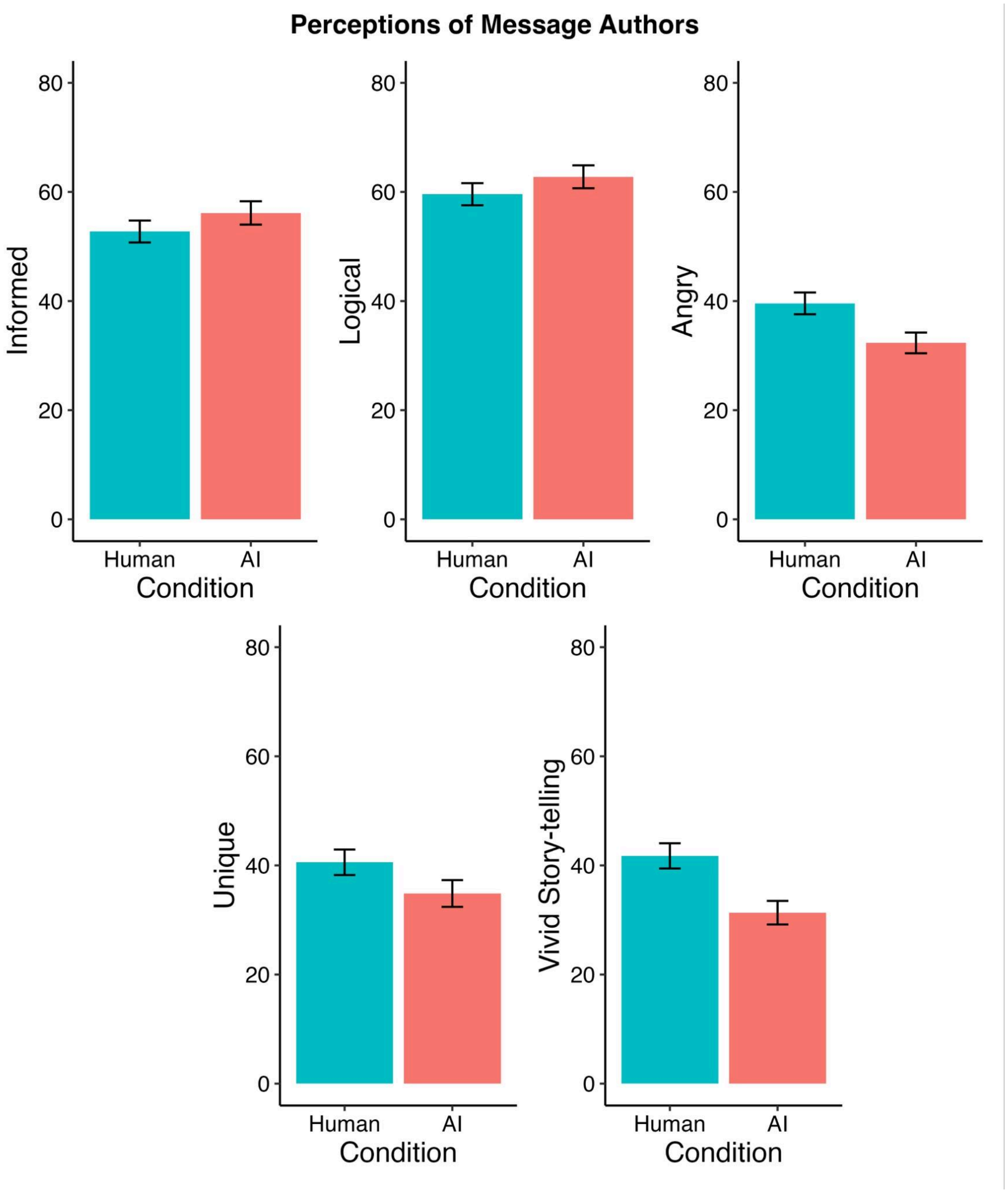

Supplementary Figure 2. Different Perceptions of Human and AI Authors in Studies 1 and 2.
Note. Y-axes represent the average perception of how informed, logical, angry, unique, and vivid in story-telling narrative, respectively, Human and AI messages were rated by participants to be (scaled from 0 - 100, 100 = highest rating). Data are presented as mean values with 95% confidence intervals. $N_{Studies\ 1\ and\ 2}$ *= 3,219 participants.*

Distribution of the Messages' Persuasiveness in Studies 1 and 2

The histograms in Supplementary Figure 3 tabulate the distribution of the persuasiveness (the difference between participants' post-treatment and pre-treatment policy support) of the messages in Studies 1 and 2. Though our studies are underpowered to provide precise estimates, overall, the variance of the persuasiveness of the messages appears to be somewhat greater among human-generated messages than LLM-generated messages.

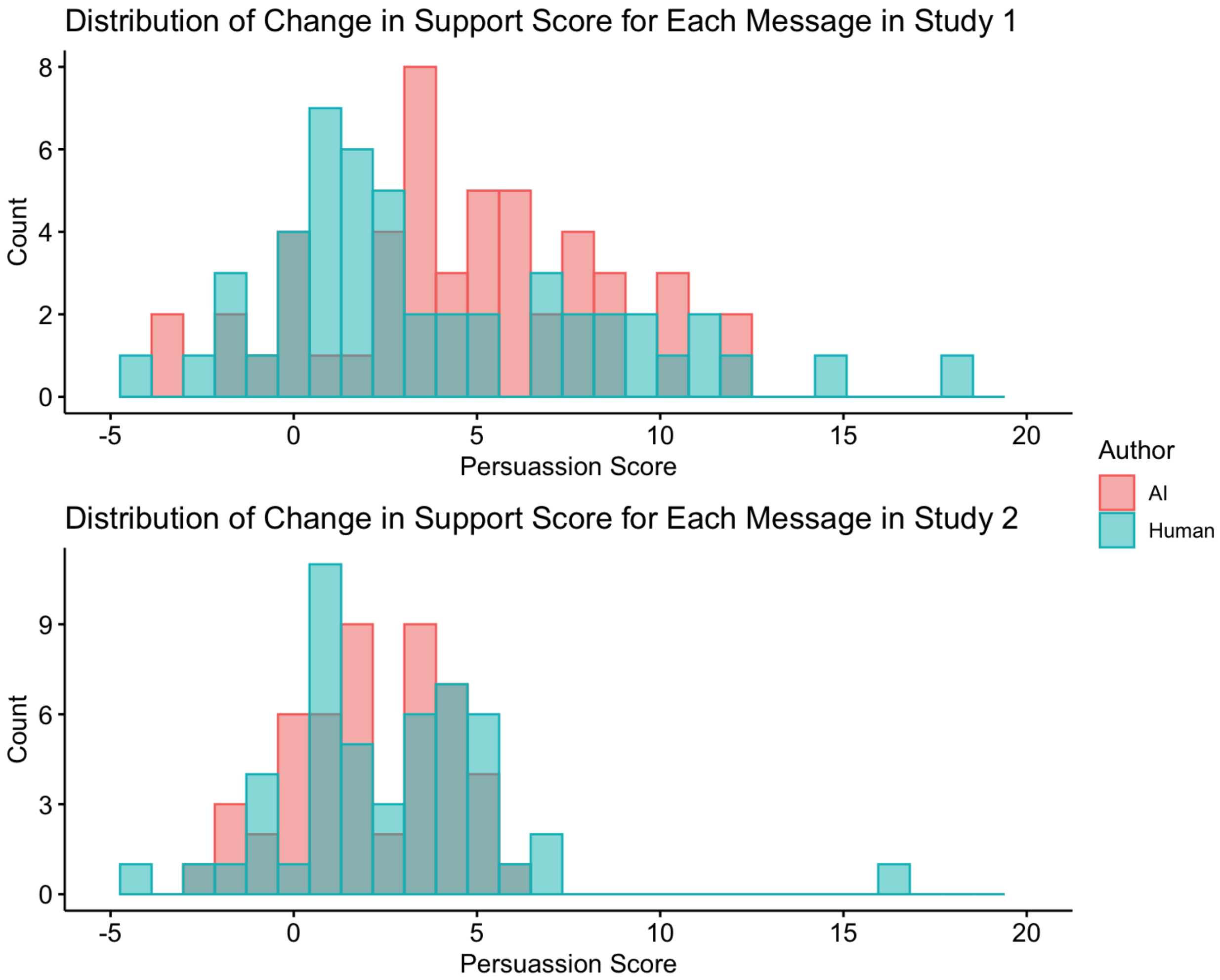

Supplementary Figure 3. Distribution of Persuasion Score of the Messages in Studies 1 and 2. *Note. X-axes represent the change in policy support from pre- to post-treatment (i.e., "persuasion scores") for participants in both the AI- and Human-author conditions (scaled from -100 to 100). Negative scores indicate a decrease in support following treatment, whereas positive scores indicate an increase in support following treatment. Y-axes represent the percent frequency of participant persuasion scores.* $N_{Study\ 1}$ *=1,203 participants,* $N_{Study\ 2}$ *= 2,016 participants.*

Distribution of Pre-Treatment Levels of Support for Policies

In the studies, we intended to test the persuasion effects on policies that vary in their levels of polarization. For Study 1, we chose a policy that we expected to be relatively favorable to demonstrating persuasion effects. We chose the policy of a smoking ban because this policy is not highly polarized, nor widely discussed. For Study 2, we sought to establish the robustness of the persuasion effects. We chose the policy of an assault weapon ban because this policy is a more polarized and widely discussed issue[1]. For Study 3, we sought to more thoroughly establish the robustness of our findings. Because relying on multiple treatments on different topics can improve causal inference compared to using just one treatment and topic[2], we chose several, different policies that are highly polarized and widely discussed: a carbon tax, an increased child tax credit, a parental leave program, and automatic voter registration.

To verify our assumption that the issue in Study 1 (a smoking ban) is not very polarized and that Studies 2 and 3 are more polarized, we reviewed the mean scores of the pre-treatment level of support for these policies by participants' party affiliations and inspected the distributions.

In Study 1, the mean level of pre-treatment support is 62.9 for self-reported Democrats, 52.1 for self-reported Independents, and 54.7 for self-reported Republicans with an 8.2 point gap between self-reported Democrats and Republicans. However, in Study 2, the mean pre-treatment support levels were 73.8 for self-reported Democrats, 49.6 for self-reported Independents, and 33.8 for self-reported Republicans, indicating a larger gap of 39.9 points between self-reported Democrats and Republicans. Similarly, in Study 3, when collapsed across all 4 issues, the mean pre-treatment support levels were 77.2 for self-reported Democrats, 61.4 for self-reported Independents, and 46.6 for self-reported Republicans, further illustrating a notable partisan gap, here at 30.6 points between self-reported Democrats and Republicans.

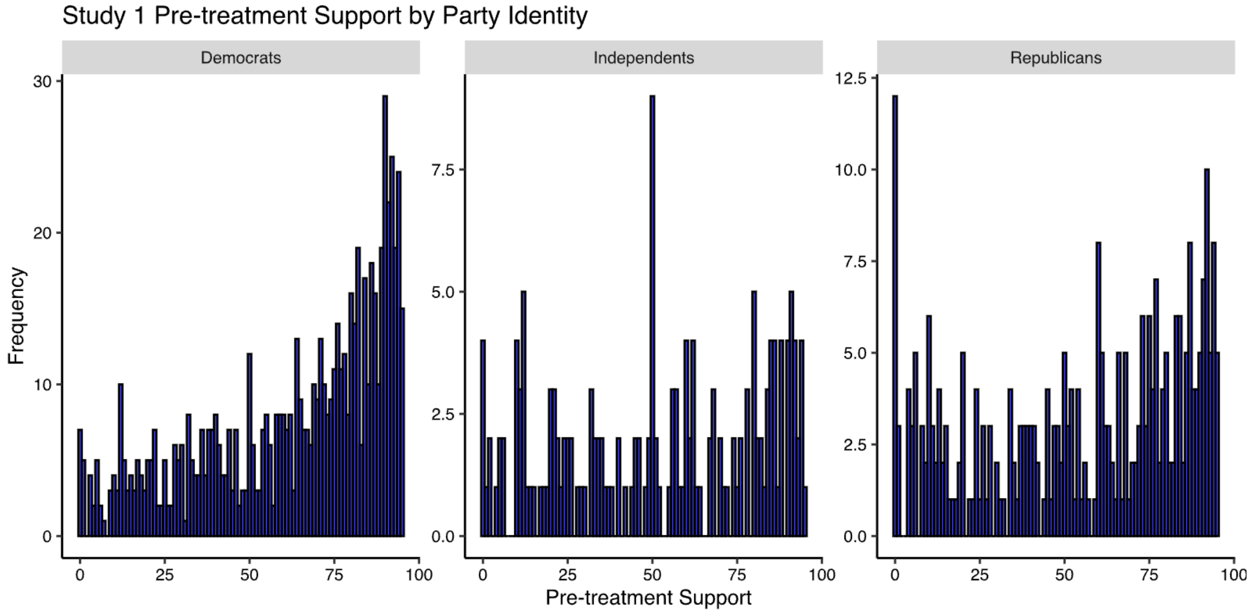

Supplementary Figure 4. Distribution of Pre-treatment Support by Participants' Partisan Identity in Study 1.

Note. X-axes represent participant pre-treatment policy support scores for a smoking ban (scaled from 0 - 100, 100 = highest level of support). Y-axes represent the frequency of pre-treatment policy support scores. $N_{Study\ 1}$ *=1,203 participants.*

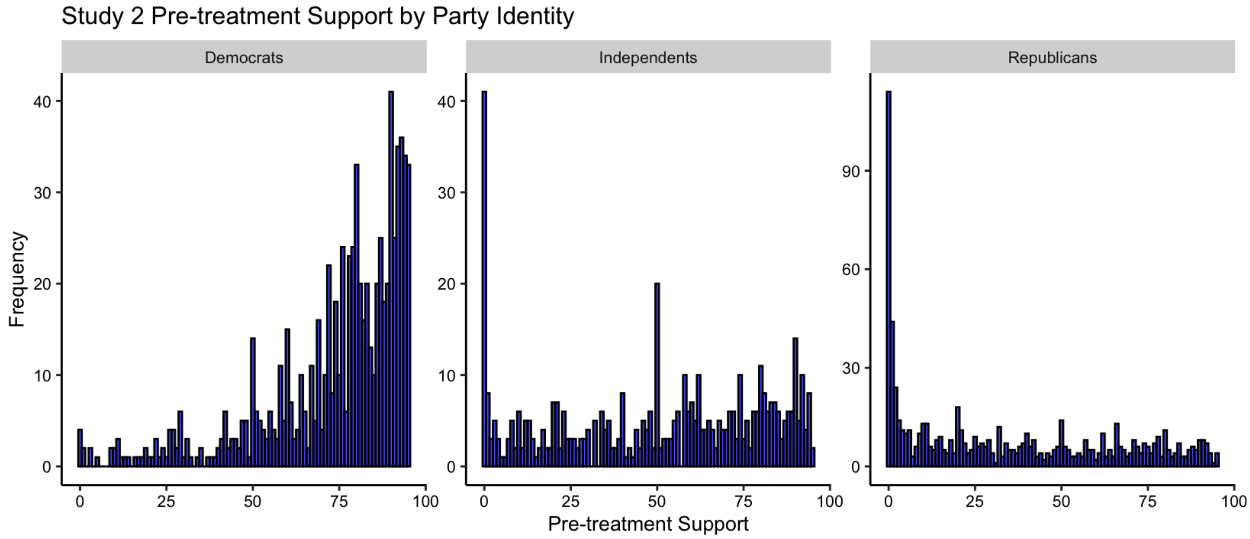

Supplementary Figure 5. Distribution of Pre-treatment Support by Participants' Party Identity in Study 2.

Note. X-axes represent participant pre-treatment policy support scores for an assault weapon ban (scaled from 0 - 100, 100 = highest level of support). Y-axes represent the frequency of pre-treatment policy support scores. $N_{Study\ 2}$ *= 2,016 participants.*

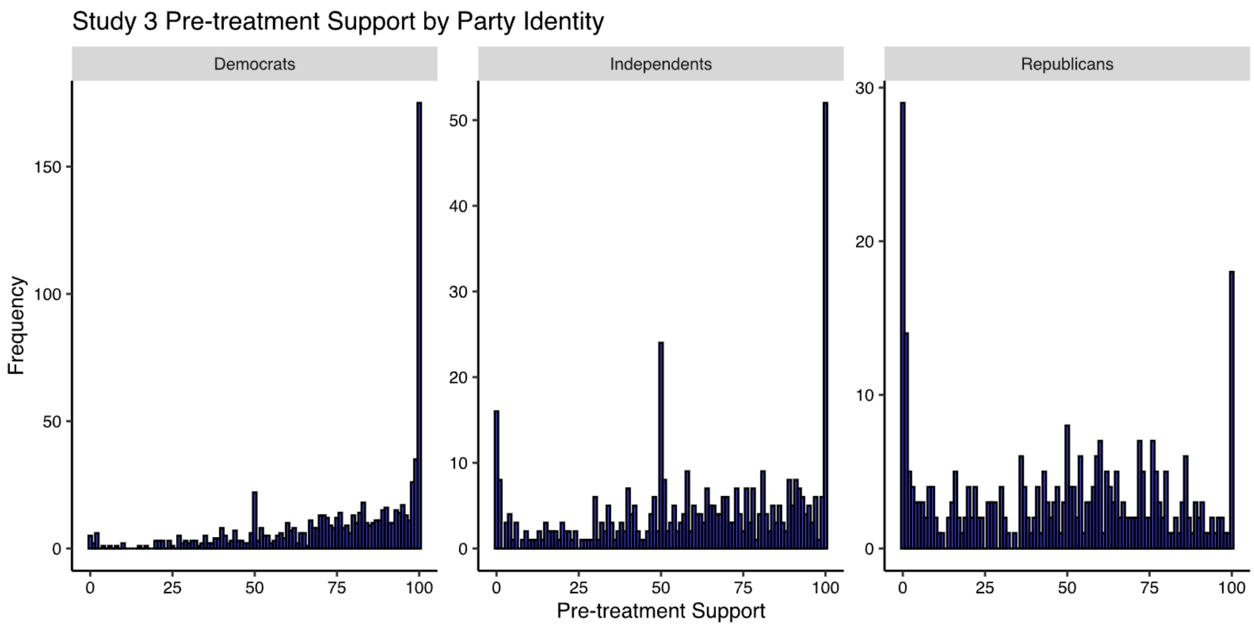

Supplementary Figure 6. Distribution of Pre-treatment Support by Participants' Party Identity in Study 3.

Note. X-axes represent participant pre-treatment policy support scores collapsed across all four issues (i.e., a carbon tax, a child tax credit, a parental leave program, automatic voter registration; scaled from 0 - 100, 100 = highest level of support). Y-axes represent the frequency of pre-treatment policy support scores. $N_{Study\ 3}$ *=1,610 participants.*

Study 3's Issue-Specific Results

We examined the persuasion effect of AI-generated messages on each of the four issues in Study 3.

Analysis Strategy

For each topic, we restricted the sample to participants in the corresponding topic condition (Carbon Tax: N = 401, Child Tax Credit: N = 402, Parental Leave Program: N = 390, and Automatic Voter Registration: N = 417). We regressed the post-treatment policy on the treatment condition, controlling for pre-treatment policy support.

Results

We found significant persuasion effects for three of the four issues. Participants in the LLM condition supported a carbon tax more than participants in the Control condition ($b = 3.74$, CI = [2.34, 5.14], $p < .001$). Participants in the LLM condition supported a child tax credit more than participants in the Control condition ($b = 5.03$, CI = [3.11, 6.96], $p < .001$). Participants in the LLM condition supported a parental leave program more than participants in the Control condition ($b = 2.39$, CI = [.82, 3.96], $p = .003$). The only issue for which we did not obtain a significant effect was automatic voter registration. While participants in the LLM condition supported automatic voter registration more than participants in the Control condition, this effect was not significant ($b = 1.45$, CI = [-.31, .321], $p = .107$).

Covariate Balance Checks

In Study 1, a one-way ANOVA revealed that the pre-treatment level of support does not differ by condition effect, $F(3, 1199) = 1.756$, $p = .154$.

In Study 2, a one-way ANOVA revealed that the pre-treatment level of support differed by condition effect, $F(3, 2012) = 3.289$, $p = .020$. Post-hoc comparisons using the Tukey HSD test indicated that the mean score for the Human condition was significantly higher than that for the Control condition (Mean difference = 6.24, $p = .011$). However, no other pairwise comparisons were statistically significant: Control vs. LLM (Mean difference = -2.45, $p = .626$), Human-in-the-Loop vs. LLM (Mean difference = 1.25, $p = .928$), Human vs. AI (Mean difference = 3.80, p = .246), Human-in-the-Loop vs. Control (Mean difference = 3.70, $p = .263$), and Human vs. Human-in-the-Loop (Mean difference = 2.54, $p = .595$).

In Study 3, four t-tests showed no significant difference in means between the Control and LLM conditions for all four policies: $t(399) = -0.042$, $p = .966$ for the carbon tax, $t(383.44) = -0.633$, $p = .527$ for the paid parental-leave program, $t(399.98) = 0.522$, $p = .602$ for the child tax credit and $t(414.12) = 0.397$, $p = .691$ for the automatic voter registration policy.

Though participants in the Human and Control conditions in Study 2 differ in their pre-treatment level of support for the policy, we consider this difference likely a result of random chance. More importantly, this difference does not impact the interpretations of our results, as these pre-treatment differences were controlled for in the analyses specifically to address the possibility of randomization failure. Additionally, as shown above, in parallel models predicting the change in participants' support for the policies (post-treatment support-pre-treatment support), the results are substantively the same, suggesting the robustness of our results.

Supplementary Discussion

Additional Literature

In the main text, we discuss that, broadly speaking, research has demonstrated that automated systems can disseminate existing human-created misinformation (e.g., by retweeting), but none shows that LLM can create original materials to influence political opinions. Below is a table summarizing related research.

Supplementary Table 12. Summary of Related Research

Paper	Study (n)	Experimental Design	Outcomes	Messages Authored By AI	Demonstrated political persuasion
Jang, W., Chun, J. W., Kim, S., & Kang, Y. W. (2023). The effects of anthropomorphism on how people evaluate algorithm-written news. Digital Journalism, 11(1), 103-124.	1 (n = 125)	Participants read and evaluated a series of news articles that was determined by a 3 (source type: algorithm-written news vs humanized algorithm-written news vs human-written news; between-subjects) × 3 (sport covered: baseball vs football vs basketball; within-subjects) design.	1. Formative message credibility 2. Reflective message credibility 3. Emotional involvement 4. Article liking 5. Behavioral consequences (intentions)	Mostly, but constant across conditions. Only the description of the source was manipulated.	No. Policy attitudes (political or otherwise) were not measured.
	2 (n = 202)	Participants read and evaluated a series of news articles that was determined by a 2 (source type: non-humanized algorithm-written news vs humanized algorithm-written news; between-subjects) × 2 (relationship with human writers: partner vs servant; between-subjects) x 2 (news content: data driven vs non-data driven; within-subjects) design.	1. Formative message credibility 2. Reflective message credibility 3. Emotional involvement 4. Article liking 5. Behavioral consequences (intentions)	Mostly, but constant across conditions. Only the description of the source was manipulated.	No, policy attitudes were not measured.

Paper	Study (n)	Experimental Design	Outcomes	Messages Authored By AI	Demonstrated political persuasion
Jang, W., Kwak, D. H., & Bucy, E. (2022). Knowledge of automated journalism moderates evaluations of algorithmically generated news. New Media & Society, 14614448221142534.	1 (n = 101)	Participants read and evaluated a series of news articles that was determined by a 2 (authorship type: human vs algorithm; between-subjects) × 3 (sport covered: baseball vs football vs basketball; within-subjects) design.	1. News credibility 2. Reading satisfaction 3. Narrative immersion 4. Emotional involvement 5. Article liking 6. Behavioral intentions to further read or return to the news source at a later time	Mostly, but constant across conditions. Only the description of the source was manipulated.	No, policy attitudes were not measured.
	2 (n = 220)	Participants read and evaluated a series of news articles that was determined by a 3 (authorship type: human vs human-like algorithm vs machine-like algorithm; between-subjects) × 3 (sport covered: baseball vs football vs basketball; within-subjects) design.	1. News credibility 2. Reading satisfaction 3. Narrative immersion 4. Emotional involvement 5. Article liking 6. Behavioral intentions to further read or return to the news source at a later time	Mostly, but constant across conditions. Only the description of the source was manipulated.	No, policy attitudes were not measured.

Paper	Study (n)	Experimental Design	Outcomes	Messages Authored By AI	Demonstrated political persuasion
Kim, T. W., & Duhachek, A. (2020). Artificial intelligence and persuasion: A construal-level account. Psychological science, 31(4), 363-380.	1a (n = 100)	Participants evaluated whether a series of actions were consistent with either low- or high-level construal. A 2 condition (agent: human vs artificial agent; between-subjects) design determined who conducted the action.	Behavioral identification (i.e., extent to which an action is perceived as consistent with either low- or high-level construal)	No, there were no messages in this study.	No, policy attitudes were not measured.
	1b (n = 191)	Participants evaluated whether some statements were appropriate. A 2 (agent: human vs artificial agent; between-subjects) x 2 (action description: high vs low construal; between-subjects) design determined the agent conducting the action and whether it was described in high- or low-construal terms.	Appropriateness of statements	No, there were no messages in this study.	No, policy attitudes were not measured.
	1c (n = 119)	Participants received a recommendation from a robot. A 2 condition (agent: learning artificial agent vs a non-learning artificial agent; between-subjects) design determined the description of the robot to the participants.	1. Inference about the robot's superordinate goals<br>2. Focus on desirability of recommended product<br>3. Extent of thoughts about robot's creator<br>4. Trust, likability, and expertise of robot	No (per communication with lead author). Also, they are constant across conditions. Only the description of the source was manipulated.	No, policy attitudes were not measured.

Paper	Study (n)	Experimental Design	Outcomes	Messages Authored By AI	Demonstrated political persuasion
Kim, T. W., & Duhachek, A. (2020). Artificial intelligence and persuasion: A construal-level account. Psychological science, 31(4), 363-380.	2 (n = 324)	Participants received a medical recommendation from a website. A 2 (agent: human vs artificial agent; between-subjects) x 2 (message: high vs low construal; between-subjects) design whether participants were told they would receive the advice from a doctor or a robot and whether the message was described in high- or low-construal terms.	Willingness to apply sunscreen	No (per communication with lead author). Also, they are constant across conditions. Only the description of the source was manipulated.	No, policy attitudes were not measured.
	3 (n = 234)	Participants received a message advertising a gym from Amazon's Alexa. A 2 (agent: learning vs non-learning artificial agent; between-subjects) x 2 (message: high vs low construal; between-subjects) design determined whether Alexa was described as having learning abilities and whether the message was described in high- or low-construal terms	1. Inference about the robot's superordinate goals 2. Intention to visit gym (persuasive effectiveness) 3. Trust, likability, and expertise of robot	No (per communication with lead author). Also, they are constant across conditions. Only the description of the source was manipulated.	No, policy attitudes were not measured.

Paper	Study (n)	Experimental Design	Outcomes	Messages Authored By AI	Demonstrated political persuasion
Kim, T. W., & Duhachek, A. (2020). Artificial intelligence and persuasion: A construal-level account. Psychological science, 31(4), 363-380.	4 (n = 100)	Participants received a message advertising a gym from a robot. A 2 condition (message: high vs low construal; between-subjects) design determined whether the message was described in high- or low-construal terms	Intention to visit gym (persuasive effectiveness)	No (per communication with lead author). Also, they are constant across conditions. Only the description of the source was manipulated.	No, policy attitudes were not measured.
	5 (n = 400)	Participants received a message advertising a product from a recommendation specialist. A 2 (agent: human vs artificial agent; between-subjects) x 2 typicality (typical vs atypical; between-subjects) x 2 (message: high vs low construal; between-subjects) design whether participants were told they would receive the message from a human or a robot, whether the source of the message was typical or atypical, and whether the message was described in high- or low-construal terms.	1. Intention to use tea tree oil and soy milk (persuasion) 2. Message fit	No (per communication with lead author). Also, they are constant across conditions. Only the description of the source was manipulated.	No, policy attitudes were not measured.

Paper	Study (n)	Experimental Design	Outcomes	Messages Authored By AI	Demonstrated political persuasion
Ischen, C., Araujo, T., van Noort, G., Voorveld, H., & Smit, E. (2020). "I am here to assist you today": The role of entity, interactivity and experiential perceptions in chatbot persuasion. Journal of Broadcasting & Electronic Media, 64(4), 615-639.	1 (n = 242)	Participants evaluated an interaction they had about a recommendation for health insurance. The interaction was determined by a 2 (communication source: chatbot vs website; between-subjects) × 2 (recommendation origin: human expert vs algorithm; between-subjects) design.	1. Recommendation adherence<br>2. Attitude toward the medium<br>3. Attitude toward the recommendation<br>4. Attitude toward the health insurance company	No, the script for the interaction was not written by AI/algorithms (according to email correspondence with the authors).	No, policy attitudes were not measured.

Paper	Study (n)	Experimental Design	Outcomes	Messages Authored By AI	Demonstrated political persuasion
Bail, C. A., Argyle, L. P., Brown, T. W., Bumpus, J. P., Chen, H., Hunzaker, M. F., ... & Volfovsky, A. (2018). Exposure to opposing views on social media can increase political polarization. Proceedings of the National Academy of Sciences, 115(37), 9216-9221.	1 (n = 1,220)	The 2 (treatment vs control; between-subjects) x 2 (pre vs post; within-subjects) design determined whether participants were incentivized (treatment) or not (control) to follow and read messages from a bot that retweeted tweets from accounts that opposed their own political leanings. Political attitudes were measured before and after the treatment.	Ideological polarization	No, the messages were retweeted by a bot but authored by humans.	Yes
Bessi, A., & Ferrara, E. (2016). Social bots distort the 2016 US Presidential election online discussion. First monday, 21(11-7).	Not an experiment (observational study of the language used by bot vs human Twitter accounts)				
Forelle, M., Howard, P., Monroy-Hernández, A., & Savage, S. (2015). Political bots and the manipulation of public opinion in Venezuela. arXiv.	Not an experiment (observational study of the prevalence of bots in retweeting and pretending to be Venezuelan politicians)				

Assumption Checks

We did not check multicollinearity as it was unnecessary for an experiment where the experimental condition variable was manipulated.

We conducted several analyses to test the normality and equal variances assumptions of the analyses. In Study 1, the Shapiro-Wilk normality test reveals that the assumptions are violated in Models 1 (W = 0.83, $p < .001$), 2 (W = 0.87, $p < .001$), and 3 (W = 0.89, $p < .001$). Additionally, the studentized Breusch-Pagan tests reveal that homoscedasticity assumptions are violated for Models 1 (BP = 58.50, $p < .001$), 2 (BP = 44.70, $p < .001$), and 3 (BP = 37.25, $p < .001$).

In Study 2, the Shapiro-Wilk normality test reveals that the assumptions are violated in Models 1 (W = 0.82, $p < .001$), 2 (W = 0.83, $p < .001$), and 3 (W = 0.85, $p < .001$). Additionally, the studentized Breusch-Pagan tests reveal that homoscedasticity assumptions are violated for Model 1 (BP = 12.66, $p = .013$), but not for Models 2 (BP = 1.01, $p = .798$) or 3 (BP = 1.19, $p = .551$).

In Study 3, the Shapiro-Wilk normality test reveals that the assumptions are violated in Models 1 (W = 0.80, $p < .001$), 2 (W = 0.72, $p < .001$), 3 (W = 0.75, $p < .001$), and 4 (W = 0.65, $p < .001$). Additionally, the studentized Breusch-Pagan tests reveal that homoscedasticity assumptions are violated for all models: Model 1 (BP = 20.15, $p < .0001$), Model 2 (BP = 14.39, $p = .001$), Model 3 (BP = 26.16, $p < .001$), and Model 4 (BP = 15.19, $p = .001$).

However, due to the Central Limit Theorem, this violation is unlikely a substantial concern for the validity of our results, given the current studies' use of large sample sizes[3]. Additionally, reestimating all of the models using Huber-White estimators reveals substantively the same, as shown in Supplementary Table 13. Therefore, overall, the violations of the assumptions of the statistical models are unlikely to invalidate our results.

Supplementary Table 13. Models Re-Estimated Using Huber-White Estimators

		b	S.E.	*p*	95% CI			
Study 1 Model 2	(Intercept)	0.56	0.84	0.504	[	-1.09	2.21	]
	Pre-treatment DV	1.00	0.01	<.001	[	0.97	1.02	]
	Human-in-the-Loop condition	5.04	0.86	<.001	[	3.36	6.73	]
	AI condition	3.62	0.71	<.001	[	2.23	5.01	]
	Human condition	3.36	0.80	<.001	[	1.80	4.93	]
Study 1 Model 2	(Intercept)	3.89	1.32	0.003	[	1.30	6.47	]
	Pre-treatment DV	1.00	0.01	<.001	[	0.97	1.03	]
	Human-in-the-Loop condition	1.68	1.05	0.108	[	-0.37	3.73	]
	AI condition	0.26	0.91	0.779	[	-1.54	2.05	]
Study 1 Model 3	(Intercept)	3.71	1.42	0.009	[	0.92	6.50	]
	Pre-treatment DV	1.00	0.02	<.001	[	0.97	1.04	]
	Human-in-the-Loop condition	1.45	0.97	0.133	[	-0.44	3.35	]
Study 2 Model 1	(Intercept)	-2.41	0.38	<.001	[	-3.15	-1.66	]
	Pre-treatment DV	1.05	0.01	<.001	[	1.04	1.06	]
	Human-in-the-Loop condition	2.35	0.51	<.001	[	1.34	3.35	]
	AI condition	1.81	0.53	0.001	[	0.78	2.84	]
	Human condition	2.35	0.53	<.001	[	1.32	3.39	]
Study 2 Model 2	(Intercept)	-0.41	0.66	0.536	[	-1.71	0.89	]
	Pre-treatment DV	1.05	0.01	<.001	[	1.04	1.07	]
	Human-in-the-Loop condition	0.01	0.62	0.984	[	-1.21	1.23	]
	AI condition	-0.52	0.63	0.410	[	-1.76	0.72	]
Study 2 Model 3	(Intercept)	-1.45	0.55	0.009	[	-2.53	-0.37	]
	Pre-treatment DV	1.06	0.01	<.001	[	1.05	1.08	]
	Human-in-the-Loop condition	0.52	0.62	0.402	[	-0.70	1.74	]
Study 3 Model 1	(Intercept)	0.30	0.85	0.727	[	-1.38	1.97	]
	Pre-treatment DV	1.00	0.01	<.001	[	0.98	1.03	]
	AI condition	3.74	0.71	<.001	[	2.34	5.14	]
Study 3 Model 2	(Intercept)	1.10	1.40	0.432	[	-1.65	3.85	]
	Pre-treatment DV	0.99	0.02	<.001	[	0.96	1.03	]
	AI condition	2.39	0.80	0.003	[	0.81	3.97	]
Study 3 Model 3	(Intercept)	0.92	1.14	0.420	[	-1.32	3.17	]
	Pre-treatment DV	0.99	0.02	<.001	[	0.96	1.03	]
	AI condition	4.13	0.80	<.001	[	2.55	5.70	]
Study 3 Model 4	(Intercept)	-0.09	0.89	0.917	[	-1.85	1.66	]
	Pre-treatment DV	1.00	0.01	<.001	[	0.98	1.02	]
	AI condition	1.32	0.70	0.062	[	-0.07	2.70	]

Note. All models are linear regression models estimated using Huber-White estimators without adjustments. S.E. refers to standard error of the estimates. All results are two-tailed.

References

1. Alfonseca, K. There have been more mass shootings than days in 2023, database shows. Retrieved from: https://abcnews.go.com/US/mass-shootings-days-2023-database-shows/story?id=96609874 (2023).
2. Fong, C., & Grimmer, J. Causal inference with latent treatments. *American Journal of Political Science*, **67(2)**, 374-389 (2023).
3. Lumley, T., Diehr, P., Emerson, S., & Chen, L. The Importance of the Normality Assumption in Large Public Health Data Sets. *Annual Review of Public Health*, **23(1)**, 151-169 (2002).
