# Supplementary material for: LLM-generated messages can persuade humans on policy issues
Source: Nat Commun. 2025 Jul 1;16:6037. doi: 10.1038/s41467-025-61345-5 (PMC12215518; doi:10.1038/s41467-025-61345-5)
Supplement: Supplementary file 2 — Reporting Summary [file 41467_2025_61345_MOESM2_ESM.pdf]

nature portfolio

Corresponding author(s): Hui Bai, Robb Willer

Last updated by author(s): Apr 1, 2025

Reporting Summary

Nature Portfolio wishes to improve the reproducibility of the work that we publish. This form provides structure for consistency and transparency in reporting. For further information on Nature Portfolio policies, see our Editorial Policies and the Editorial Policy Checklist.

Statistics

For all statistical analyses, confirm that the following items are present in the figure legend, table legend, main text, or Methods section.

n/a	Confirmed	
☐	☒	The exact sample size (*n*) for each experimental group/condition, given as a discrete number and unit of measurement
☐	☒	A statement on whether measurements were taken from distinct samples or whether the same sample was measured repeatedly
☐	☒	The statistical test(s) used AND whether they are one- or two-sided<br>*Only common tests should be described solely by name; describe more complex techniques in the Methods section.*
☐	☒	A description of all covariates tested
☐	☒	A description of any assumptions or corrections, such as tests of normality and adjustment for multiple comparisons
☐	☒	A full description of the statistical parameters including central tendency (e.g. means) or other basic estimates (e.g. regression coefficient) AND variation (e.g. standard deviation) or associated estimates of uncertainty (e.g. confidence intervals)
☐	☒	For null hypothesis testing, the test statistic (e.g. F, t, r) with confidence intervals, effect sizes, degrees of freedom and P value noted<br>*Give P values as exact values whenever suitable.*
☐	☒	For Bayesian analysis, information on the choice of priors and Markov chain Monte Carlo settings
☐	☒	For hierarchical and complex designs, identification of the appropriate level for tests and full reporting of outcomes
☐	☒	Estimates of effect sizes (e.g. Cohen's d, Pearson's r), indicating how they were calculated

Our web collection on statistics for biologists contains articles on many of the points above.

Software and code

Policy information about availability of computer code

Data collection	No data collection code was used and all studies were collected using Qualtrics online survey software. Individual Qualtrics survey files can be accessed at osf.io/8yxvr
Data analysis	All analysis codes written in R, and all analyses were conducted in RStudio 2022.07.2 Build 576. Statistical packages used in data analysis are defined in individual R scripts, which can be accessed at osf.io/8yxvr

For manuscripts utilizing custom algorithms or software that are central to the research but not yet described in published literature, software must be made available to editors and reviewers. We strongly encourage code deposition in a community repository (e.g. GitHub). See the Nature Portfolio guidelines for submitting code & software for further information.

Data

Policy information about availability of data

All manuscripts must include a data availability statement. This statement should provide the following information, where applicable:

- Accession codes, unique identifiers, or web links for publicly available datasets
- A description of any restrictions on data availability
- For clinical datasets or third party data, please ensure that the statement adheres to our policy

Anonymized data generated in all studies have been deposited in the following OSF repository: https://doi.org/10.17605/osf.io/8yxvr. The raw study data are

protected and are not available due to data privacy laws.

Research involving human participants, their data, or biological material

Policy information about studies with human participants or human data. See also policy information about sex, gender (identity/presentation), and sexual orientation and race, ethnicity and racism.

Reporting on sex and gender	For all studies, gender was determined based on self-identification of participants. We did not consider or pre-register any analyses disaggregating the data by participant's sex or gender, as it was not a theoretically relevant variable to our research questions and recent research finds the persuasive effects of political messages are similar for participants of different genders (see Anderson-Nilsson & Clayton, 2021, as cited in our manuscript). See manuscript for reporting of gender demographics in each of our study samples.
Reporting on race, ethnicity, or other socially relevant groupings	Participant race was determined by self-report in accordance with the following .survey response options: White / Caucasian, Black / African- American, Latino / Hispanic, Asian / Asian-American, Other. See manuscript for reporting of racial demographics in each of our study samples.
Population characteristics	The characteristics of the participants are described in detail in the Methods section of the manuscript.
Recruitment	Participants were recruited via the online sample provider platforms Prolific.com and CloudResearch. Though self-selection bias may occur, the authors could not think of how such biases may impact our experimental results.
Ethics oversight	All studies were approved by the Institutional Review Board at Stanford University (Protocol ID: 32506).

Note that full information on the approval of the study protocol must also be provided in the manuscript.

Field-specific reporting

Please select the one below that is the best fit for your research. If you are not sure, read the appropriate sections before making your selection.

☐ Life sciences ☒ Behavioural & social sciences ☐ Ecological, evolutionary & environmental sciences

For a reference copy of the document with all sections, see nature.com/documents/nr-reporting-summary-flat.pdf

Behavioural & social sciences study design

All studies must disclose on these points even when the disclosure is negative.

Study description	All studies were quantitative, within-between-subjects online survey experiments.
Research sample	In Study 1, after applying exclusion criteria (see below), our final sample was 1,203 participants. 567 self-identified as female and 631 as male. The mean age was 38.37 (SD = 13.12). 632 participants reported having a college-degree. 927 identified as White / Caucasian, 75 as Black / African-American, 85 as Asian / Asian-American, 69 as Latino / Hispanic, 42 as "other". 551 participants identified as Democrats, 219 as Republicans, and 377 as Independents or other. 51 participants reported having no political affiliation and demographic information from 5 participants are missing.<br><br>In study 2, after applying exclusion criteria, our final sample was 2,016 participants. 987 self-identified as female, 995 as male, 11 as non-binary, and 23 declined to report their gender. The mean age was 40.31 (SD = 13.73). 1,124 participants reported having a college-degree. 1,530 identified as White / Caucasian, 161 as Black / African-American, 69 as Latino / Hispanic, 135 as Asian / Asian-American, 83 as being mixed-race, and 36 as "other" (2 participants declined to report their race). 780 participants identified as Democrats, 760 as Republicans, and 476 as Independents.<br><br>In study 3, after applying exclusion criteria, our final sample was 1,610 participants. 828 self-identified as female and 782 as male. The mean age was 44.04 (SD = 15.37). 876 participants reported having a college degree. 1,233 identified as White / Caucasian, 187 as Black / African-American, 85 as Latino / Hispanic, 80 as Asian / Asian-American, and 25 as "Other". There were 809 who self-reported as Democrats, and 333 who self-reported as Republicans. The remaining participants self-reported as Independents and others.
Sampling strategy	We recruited a convenience sample of US participants from Prolific.com for study 1, a politically balanced sample of US participants from Prolific.com and CloudResearch for Study 2, and a representative sample for Study 3 benchmarked to US racial, gender, and age demographics. These sample choices align with common social science research practices, providing a balance between accessibility and generalizability.<br><br>For study 1, we preregistered seeking a sample size of 1200 participants (300 participants per condition). Our final sample was 1203. Sensitivity power analyses suggest that this sample size provided 95% power (alpha = .05) to detect a treatment effect of b = 2.85.<br><br>For study 2, we preregistered seeking a sample size of 2000 participants (500 participants per condition). Our final sample was 2,016. Sensitivity power analyses suggest that this sample size provided 95% power (alpha = .05) to detect a treatment effect of b = 1.85.

	For study 3, we preregistered seeking a sample size of 1,600 participants (800 participants per condition). Our final sample was 1,610. Sensitivity power analyses suggest that this sample size provided 95% power (alpha = .05) to detect a treatment effect of b = 1.23.
Data collection	We used the survey software platform Qualtrics to collect data for all three studies. Participants were blind to the study design. We did not use deception. Because the study was conducted online, there was no interaction between the experimenter and participants.
Timing	Data for Study 1 were collected on November 15, 2022. We concluded data collection on Tuesday, November 15, 2022 at 11:00 PM PT and then began analysis. Any participants completing the study after this time were not considered part of our sample.<br><br>Data for Study 2 were collected from December 5, 2022 to December 14, 2022. Because we used two separate sample providers for Study 2, however, data collection ended on different dates: ee concluded data collection on December 10, 2022 at 1:09 PM PT for the Prolific sample, and December 14, 2022 at 5:53 PM PT for the CloudResearch sample and then began analysis. Any participants completing the study after these time points were not considered part of our sample.<br><br>Data for Study 3 were collected from December 24, 2022 to December 28, 2022. We began data collection on Saturday, December 24, 2022 at 9:44 PM PT, and ended data collection on Wednesday, December 28, 2022 at 9:43 AM PT and then began analysis. Any participants completing the study after this time were not considered part of our sample.
Data exclusions	In study 1, a total of 2,096 participants responded to our recruitment advertisement. Before treatment assignment, we excluded participants (i) who had missing values for any item making up the pre-treatment dependent variable (i.e., support for a smoking ban), (ii) who already reported a very high level of support for the smoking ban (response to the composite score for the smoking ban of more than 95), (iii) who failed the attention check, (iv) who dropped out of the study before treatment assignment, and (v) who indicated that they were under 18 years old. After treatment assignment, we excluded from our analysis those (vi) who had missing values for any item making up the post-treatment dependent variable (i.e., support for a smoking ban) and (vii) with the same Participant ID by keeping only the first case, leaving us with a final sample of 1,203. In total 893 participants were excluded from our analysis.<br><br>In study 2, a total of 3,541 participants responded to our recruitment advertisement. Before treatment assignment, we excluded participants (i) who had missing values for any item making up the pre-treatment dependent variable (i.e., support for an assault weapon ban), (ii) who already reported a very high level of support for an assault weapon ban (response to the composite score for an assault weapon ban of more than 95), (iii) who failed the attention check, (iv) who dropped out of the study before treatment assignment, and (v) who indicated that they were under 18 years old. After treatment assignment, we further excluded from our analysis those (vi) who had missing values for any item making up the post-treatment dependent variable (i.e., support for an assault weapon ban), and (vii) with the same Participant ID by keeping only the first case, leaving us with a final sample of 2,016 participants. In total 1525 participants were excluded from our analysis.<br><br>In study 3, a total of 1,795 participants from Prolific.com responded to our recruitment advertisement. Before treatment assignment, we excluded participants (i) who had missing values for any item making up the pre-treatment dependent variable (i.e., policy topic; see "Procedure" below), (ii) who failed the attention check, (iii) who dropped out of the study before treatment assignment, (iv) and who indicated that they were younger than 18 years old. After treatment assignment, we further excluded from our analysis those (v) with the same Participant ID by keeping only the first case. Because none of these remaining participants had missing values for any item making up the post-treatment dependent variable, we did not further exclude any participants from our analysis, leaving us with a final sample of 1,610. In total, 185 participants were excluded from our analysis.
Non-participation	Drop-outs were accounted for in our preregistered exclusion criteria (please see above).
Randomization	Participants were randomly assigned to experimental conditions.

Reporting for specific materials, systems and methods

We require information from authors about some types of materials, experimental systems and methods used in many studies. Here, indicate whether each material, system or method listed is relevant to your study. If you are not sure if a list item applies to your research, read the appropriate section before selecting a response.

Materials & experimental systems

n/a	Involved in the study	
☒	☐	Antibodies
☒	☐	Eukaryotic cell lines
☒	☐	Palaeontology and archaeology
☒	☐	Animals and other organisms
☒	☐	Clinical data
☒	☐	Dual use research of concern
☒	☐	Plants

Methods

n/a	Involved in the study	
☒	☐	ChIP-seq
☒	☐	Flow cytometry
☒	☐	MRI-based neuroimaging

Plants

Seed stocks	N/A
Novel plant genotypes	N/A
Authentication	N/A
